# The effect of proactive *versus* reactive treatment of hypotension on postoperative disability and outcome in surgical patients under anaesthesia (PRETREAT): clinical trial protocol and considerations

**DOI:** 10.1016/j.bjao.2024.100262

**Published:** 2024-02-29

**Authors:** Matthijs Kant, Wilton A. van Klei, Markus W. Hollmann, Denise P. Veelo, Teus H. Kappen, The PRETREAT study group, Eline de Klerk, Lisette Vernooij, Luuk C. Otterspoor, Geert-Jan E. Cromheecke, Marlous Huijzer, Jannie Witziers, Lotte E. Terwindt, Tim Bastiaanse, Rogier V. Immink, Magnus Strypet, Niek H. Sperna Weiland, Marije Wijnberge, Marc G.H. Besselink, Lisette M. Vernooij, Yvonne C. Janmaat, Annemarie Akkermans

**Affiliations:** 3 Department of Anaesthesiology, Amsterdam University Medical Centre, Location University of Amsterdam, Amsterdam, The Netherlands; 4 Department of Anaesthesiology, University Medical Centre Utrecht, Utrecht, The Netherlands; 5 Department of Cardiology and Department of Intensive Care Medicine, Catharina Hospital, Eindhoven, The Netherlands; 1 Department of Anaesthesiology, University Medical Centre Utrecht, Utrecht, The Netherlands; 2 Department of Anaesthesiology, Amsterdam University Medical Centre, Location University of Amsterdam, Amsterdam, The Netherlands

**Keywords:** blood pressure, intraoperative hypotension, myocardial injury, organ injury, renal injury

## Abstract

**Background:**

Intraoperative hypotension has been extensively studied for its association with adverse outcomes. However, small sample sizes and methodological issues limit the causal inference that can be drawn.

**Methods:**

In this multicentre, adaptive, randomised controlled trial, we will include 5000 adult inpatients scheduled for elective non-cardiac surgery under general or central neuraxial anaesthesia. Patients will be either randomly allocated to the intervention or care-as-usual group using computer-generated blocks of four, six, or eight, with an allocation ratio of 1:1. In the intervention arm patients will be divided into low-, intermediate-, and high-risk groups based on their likelihood to experience intraoperative hypotension, with resulting mean blood pressure targets of 70, 80, and 90 mm Hg, respectively. Anaesthesia teams will be provided with a clinical guideline on how to keep patients at their target blood pressure. During the first 6 months of the trial the intervention strategy will be evaluated and further revised in adaptation cycles of 3 weeks if necessary, to improve successful impact on the clinical process. The primary outcome is postoperative disability after 6 months measured with the World Health Organization Disability Assessment Score (WHODAS) 2.0 questionnaire.

**Ethics and dissemination:**

This study protocol has been approved by the Medical Ethics Committee of the University Medical Centre Utrecht (20–749) and all protocol amendments will be communicated to the Medical Ethics Committee. The study protocol is in adherence with the Declaration of Helsinki and the guideline of Good Clinical Practice. Dissemination plans include publication in a peer-reviewed journal.

**Clinical trial registration:**

The Dutch Trial Register, NL9391. Registered on 22 March 2021.

Intraoperative hypotension has been extensively studied for its assumed association with adverse outcomes, such as myocardial ischaemia, renal failure, and mortality.^1^, ^2^, ^3^, ^4^, ^5^, ^6^, ^7^, ^8^ Most studies, however, were observational and therefore prone to residual confounding. Moreover, the current standard of intraoperative blood pressure management is predominantly reactive: blood pressure is treated when it approaches a specified minimally acceptable threshold (e.g. a mean arterial pressure [MAP] of 65 mm Hg) or when it is rapidly decreasing. From a hazard perspective, a proactive approach seems warranted: setting higher blood pressure targets in order to intervene earlier and keep the blood pressure at a certain margin above a specified minimally acceptable threshold (see Fig 1). Currently, an MAP of 65 mm Hg is generally considered the minimally acceptable threshold, because lower blood pressures are associated with an increased risk of adverse outcomes.^9^, ^10^, ^11^ Patients at higher risk of blood pressure fluctuations should likely be kept at a higher margin to avoid any blood pressures below the minimally acceptable threshold.

**Fig 1:**
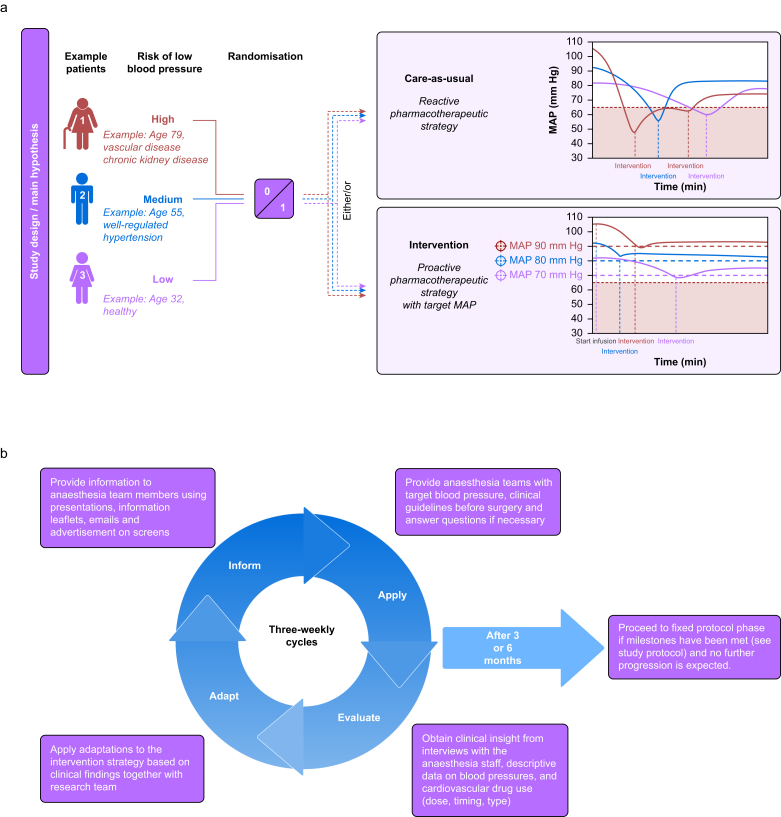
(a) Hypothesis and intervention rationale. (b) Adaptation cycles during the first 3–6 months of the trial.

Although several randomised trials have been undertaken, small sample sizes and methodological issues limit the causal inference that can be drawn.^12^^,^^13^ Designing an adequately sized randomised trial to more definitively estimate the potential adverse effects of intraoperative hypotension, however, is not straightforward. Although >75% of patients experience episodes of hypotension during surgery, its severity differs substantially and a similar degree of hypotension in two patients does not necessarily translate to identical outcomes.^9^, ^10^, ^11^ Even though this inter-individual variation is not fully understood, it is likely the result of an interplay between different pathological pathways and the combined actions of the surgical and anaesthesia teams.

In this article we discuss how the interplay between intraoperative hypotension and other perioperative factors served as a rationale underlying the design of the currently ongoing PRETREAT trial (The effect of Proactive versus REactive TREATment of hypotension on postoperative disability and outcome in surgical patients under anaesthesia). PRETREAT was designed as a multicentre, adaptive, randomised clinical trial, comparing a proactive, risk-based intraoperative blood pressure management strategy with care-as-usual (i.e. a reactive blood pressure management strategy). PRETREAT includes 5000 patients and assesses whether intraoperative hypotension indeed causes adverse patient outcomes. We provide an outline of the trial design and a more detailed explanation of the design choices.

## Methods

### Ethical approval and trial registration

This study protocol has been approved by the Medical Ethics Committee of the University Medical Centre Utrecht (20–749) and all protocol amendments will be communicated to the Medical Ethics Committee. The current version of the protocol (Version 6, 22-9-2023) is provided in Supplementary Materials 1. The study protocol is in adherence with the Declaration of Helsinki and the guideline of Good Clinical Practice. The trial was registered with The Dutch Trial Register (reference, NL9391, first registration 22 March 2021).

### Study design

This is a multicentre, adaptive, randomised controlled trial with a 1:1 allocation ratio conducted at the University Medical Centre Utrecht (UMCU) and Amsterdam University Medical Centre, both in the Netherlands. Inclusion began on 17 June 2021, with a planned 24-month duration. This paper adheres to the reporting guideline for clinical trial protocols (SPIRIT—see Supplementary Materials 2 for checklist).^14^

### Eligibility criteria

Adult inpatients undergoing elective noncardiac surgery with general anaesthesia or central neuraxial anaesthesia are eligible for inclusion. Exclusions apply to low-risk surgeries (e.g. ophthalmic, endoscopic, radiological procedures, or procedures of <30 min duration), obstetric procedures, organ transplantations, participation in conflicting clinical trials, inability to meet study requirements (e.g. legal incapacity or language barriers), and patients with an American Society of Anesthesiologists (ASA) physical status of 5. All participants are insured at both locations.

#### Explanation of the trial design choices—eligibility

Previous studies typically included high-risk patients, yet several observational studies suggest that the adverse effects and magnitude of intraoperative hypotension are not limited to high-risk patients.^2^^,^^5^, ^6^, ^7^, ^8^^,^^13^^,^^15^ Hence, our trial includes a broad patient population.

### Intervention strategy

We hypothesised that a proactive strategy can maintain blood pressure above a minimal acceptable threshold of MAP 65 mm Hg. This strategy comprises two components:

1 Target blood pressure: Patients are categorised into low-, intermediate-, and high-risk groups for intraoperative hypotension based on a hypotension risk score. Their corresponding blood pressure targets are MAP 70, 80, and 90 mm Hg, respectively.
2 Guidelines for achieving target blood pressure: A clinical guideline (see Appendix 2 of the study protocol) offers proactive treatment options to maintain target blood pressure. As no convincing evidence exists as to what is considered the optimal drug, each centre can use their preferred vasopressors, maintaining consistent drug classes for similar situations.
  The risk groups will be based on a centre-specific risk score estimating the risk of intraoperative hypotension. As no comprehensive list of risk factors is available in the current literature, we used multivariable regression analyses of historical data from each centre to estimate which risk factors are associated with intraoperative hypotension or treatment of hypotension (i.e. perioperative vasopressor use). Only risk factors available before surgery in >99% of the cases were included to enable stratified randomisation. An example of the risk score can be found in the study protocol (Supplementary Materials 1). The development of the risk score will be discussed in depth in another article. For the control group, blood pressure management follows standard clinical practice, without a predefined protocol (care-as-usual). Healthcare providers will not be informed of study participation for control group patients in order to best maintain ‘care-as-usual’.

#### Explanation of the trial design choices—intervention strategy

Intraoperative blood pressure management is complex and therefore a single drug treatment or dosage scheme intervention is not available. Striking a balance between a user-friendly and implementable strategy while accommodating clinical variations encountered in practice is crucial. To achieve the target blood pressure, a treatment recommendation was developed (see study protocol, Supplementary Materials 1). The underlying assumption for the treatment recommendation was that current clinical practice falls short in: (a) the anticipated treatment of the vasodilatory (and in a lesser way negative inotropic) side-effects of anaesthesia during or shortly after induction; and (b) the response to unanticipated decreases in blood pressure by other causes. These two shortcomings are both covered in the treatment recommendation. More details on the implementation of the strategy can be found in Supplementary Materials 1, the study protocol.

### Outcomes

The primary outcome is disability at 6 months after surgery measured by the 12-item World Health Organization Disability Assessment Score (WHODAS) 2.0 scale.^16^ The secondary outcomes that will be collected during the trial can be found in Supplementary Materials 1. All required data are routinely collected within the current standards of care including the WHODAS 2.0 and EQ-5D-5L questionnaires. All other data will be extracted from the electronic patient record through the enterprise data warehouse from each centre. The extensive use of routine clinical care data for this trial ensures minimal patient burden and promotes compliance. For an overview of the outcome assessment see Table 1.

**Table 1:** Outcome assessments of the PRETREAT trial. ∗ All required data are routinely collected within the current standards of care, including the WHODAS 2.0 and EQ-5D-5L questionnaires. See data collection for time points for data extraction.

	Study period							
			Follow-up					
Timepoint∗	Pre-surgery	During surgery	24 hours	48 hours	7 Days	30 Days	6 Months	Until discharge
Enrolment:								
Eligibility screen	X							
Written and oral information	X							
Informed consent	X							
Stratified randomisation	X							
Inform anaesthesia team	X							
Assessments:								
Determinants	X							
Process level		X						
Behavioural level		X						
Questionnaires (WHODAS 2.0 and EQ-5D-5L)	X					X	X	
Clavien–Dindo and surgical complications								X
In-hospital mortality								X
Mortality within 48 h			X	X				
All-cause mortality		X	X	X	X	X	X	X
Length of hospital stay								X
Troponin and creatinine			X	X	X			
Intensive care admission								X
Non-prophylactic antibiotics								X
Readmission in same hospital			X	X	X	X	X	
Reoperation during hospital stay								X
Haemorrhage requiring blood transfusion			X	X				
Onset cardiac complications			X	X				
In-hospital life-threatening events								X

#### Explanation of the trial design choices—outcomes

Single-organ injury may affect other organs and impose functional disabilities at the level of the individual as a whole. The primary outcome of the trial must capture these multiple, simultaneous effects. A composite endpoint seems adequate but composite endpoints require that the frequency and severity of the individual elements included are comparable.^17^ In the case of hypotension, the incidence and severity of the different organ injuries are likely not comparable. A patient-centred outcome captures the total impact of various organ injuries without the need for equal weights. The WHODAS 2.0 questionnaire is a validated and reliable measure for postoperative disability and is recommended by the Standardized Endpoints in Perioperative Medicine (STEP) initiative.^18^^,^^19^ A 5% point difference in WHODAS 2.0 score is considered a clinical relevant difference.^18^

### Participant timeline, randomisation, and blinding

Patients will be recruited and provided with study information before surgery by a trial nurse. After receiving information, they will sign informed consent forms before the randomisation process. A secure server will maintain a subject-screening and enrolment log accessible to study personnel only. Data, including the ASA score, planned operation duration, age, and surgical specialty, will be collected from medical records to determine individual risk groups (see Fig 1a). Randomisation will be done by a trial nurse using Castor EDC software, stratified for the risk group using concealed computer-generated blocks of four, six, or eight, with an allocation ratio of 1:1. Anaesthesia teams will receive randomisation results in advance by mail, telephone, and automatic pop-up in the anaesthesia system. Clinical guidelines will be available in both paper and digital formats. For a Consolidated Standard of Reporting Trials (CONSORT) flow diagram of the study see Supplementary Materials 3.

As part of routine care, participants receive WHODAS 2.0 and EuroQoL 5D-5L questionnaires three times: before surgery, at 30 days, and 6 months after surgery. Informed consent will include permission to use questionnaire results.

Both the randomisation and the assessment of the outcomes may be done by the same research team member. Hence, the assessment of the outcomes is not blinded. We will make sure that the randomisation status is obscured from the assessor's view within the workflow process. Patients are not actively blinded for the intervention, as they are able to view their intraoperative medical record. Nonetheless, the result of the randomisation will not actively be told to the patient. Caregivers cannot be blinded to the intervention because of the nature of the intervention strategy.

#### Explanation of the trial design choices—randomisation

There are many factors in the interplay between the pathophysiology of hypotension and the anaesthetic and surgical factors, resulting in a large variation of pathways that may explain the possible relation between hypotension and adverse patient outcome.

One source of this variation is the magnitude of hypotension. The depth and duration of hypotension are not ‘equipotent’ in their impact on organ injury, a shorter duration but greater decrease in blood pressure has a stronger effect on organ injury than a prolonged but smaller decrease.^15^ However, the degree of hypotension in both depth and duration may substantially differ between patients, resulting in various patterns.

Second, there is variation in procedural factors, such as the surgical stress response, tissue damage, and anaesthetic interventions that are an important direct source of organ injury.^20^^,^^21^ Conversely, these procedural factors may also cause intraoperative hypotension. In addition, patient factors must be considered, as pre-existing comorbidities may already have resulted in organ injury. A period of modest hypotension will likely have more impact on organs in those with chronic disease. At the same time, pre-existing conditions (e.g. atherosclerosis), render the patient susceptible to intraoperative hypotension and as such, hypotension can be considered as a biomarker for organ injury. This interplay is not simply confounding, but rather a complex of effect modifications. It is much more likely that all these different conditions amplify each other's impact, rather than their cumulative impact being summative.

The complex interplay between hypotension, patient and procedural factors makes randomisation less straightforward. To be able to study the causality of effects for different magnitudes of hypotension in relation to patient and procedural factors, randomisation should be stratified for magnitudes of hypotension. As the true magnitude of hypotension is not known at the time of randomisation, we use the expected risk of intraoperative hypotension (low, intermediate, high) as a substitute (see Intervention Strategy above for more details on risk classification). When randomisation is stratified for the degree to which patients are prone to suffer from hypotension, there is likely a better balance between patient and procedure risk factors within risk strata.

### Implementation

During the first 6 months of the trial, the risk-based intervention strategy will be evaluated and revised in 3-weekly adaption cycles (i.e. the adaptation phase, see Fig 1b). Based on clinical insight obtained from weekly interviews with the anaesthesia staff and descriptive blood pressure and cardiovascular drug use data, the impact of the proactive intervention strategy will be evaluated, and adjustments will be made by the research team (e.g. reducing administration of vasopressors if the intervention strategy leads to overtreatment and subsequent hypertension). If the risk-based intervention strategy is not able to reduce the relative risk of hypotension by 30%, the study will end after the adaptation phase.

When an intervention group patient is scheduled for surgery, the anaesthesia care providers will receive an e-mail and an information folder with the advised target blood pressure the day before the procedure. On the day of the procedure a research team member visits or calls the anaesthesia care providers to answer questions and provide them with the flowchart. The research team member will not be present during the surgical procedure.

#### Explanation of the trial design choices—the adaptation phase

Changing the behaviour of the anaesthesia care providers is not straightforward. Although the intervention strategy was designed for its ease of use while accommodating clinical variation, we cannot presume that the intervention strategy is easy to adhere to. Moreover, we considered successful implementation of our study even more difficult because the intervention strategy could lead to overtreatment, resulting in hypertension and subsequent complications. To foster clinical adherence, we decided to include an adaptation phase that will allow us to adjust the intervention strategy to improve its adoption by the care providers (see study protocol in Supplementary Materials 1).

### Sample size calculation

Based on the literature and the mean and standard deviation of the WHODAS 2.0 score in the participating centres, we expect a 5% point change (from 17% to 12%) in functional disability at 6 months in the intermediate-risk stratum with a 17% point within-cluster standard deviation, and a between-cluster standard deviation of 0.5% point.^13^ The sample size was increased by 20% to account for possible loss-to-follow-up. We calculated a cluster-adjusted sample size using the clusterPower package (version 0.6.111) in R software (R Foundation for Statistical Computing, Vienna, Austria).^22^ This resulted in an estimated sample size of 2248 patients in the intermediate-risk stratum, with an overall sample size of 4496 patients divided over two study groups at two centres over 2 yr of recruitment. However, because of the COVID-19 pandemic only a limited number of questionnaires were available to calculate the sample size and power (*n*=770). Because of this uncertainty we decided to raise the estimated sample size to 5000 patients resulting in 0.94 power. Both participating centres have declared that the inclusion rate is feasible.

### Statistical analysis

The complexity of the trial design requires specific considerations during the analysis phase and the interpretation of the results. The proactive strategy has a mechanistic hypothesis: (1) implementation of the treatment recommendation will change the administration of vasoactive drugs by the anaesthesia team; (2) change in behaviour results in a substantial reduction in intraoperative hypotension; and (3) reduction in hypotension improves patient outcome. Failure in any of these three steps will likely result in a failure to support the hypothesis. To protect ourselves from finding spurious results, we have added a go/no-go decision at the end of the adaptation phase: if there is no substantial reduction in intraoperative hypotension we will stop the trial, as it would be both a risk for spurious results and a waste of money to continue. The interim analyses at the end of and during the adaptation phase makes null hypothesis testing less appropriate. We will hence analyse this trial using a Bayesian framework. There are many more details to be considered, including preplanned alternative analysis and secondary outcomes, for which we refer to the study protocol in Supplementary Materials 1.

### Contamination

As the anaesthesia team becomes more experienced with the intervention strategy, it is possible that they will start applying (parts of) the intervention strategy to patients of the care-as-usual group (cross-contamination). As a result, any initial differences between the groups may converge over time. Cluster-randomisation or similar designs were considered to avoid contamination. However, a previous study showed that randomisation of anaesthesiologists may cause baseline differences explained by the different case mix of patients per anaesthesiologist.^23^ In addition, the composition of anaesthesia teams changes every day and it is therefore not possible to randomise per anaesthesiologist without achieving crossover effects. If systematic contamination occurs, the trial will be converted to a before–after design as described in the study protocol, using the pre-trial period as ‘before-period’.^24^ All required data are readily available from pre-trial patients as those data were collected as part of routine clinical care.

### Premature termination of the study

After the inclusion of 2500 patients, the effectiveness of the intervention strategy will be determined. If the patients in the intervention arm show a 5% point or more increase in functional disability after 30 days compared with the care-as-usual arm measured with the WHODAS 2.0 questionnaire, the study team will discuss whether or not to terminate the study prematurely. Termination of the trial because of insufficient effectiveness of the intervention strategy will not be considered premature termination, rather a planned termination of the trial.

### Safety

The risk of participating in this study is defined as ‘moderate’. We will cover potential harm by closely monitoring how the strategy affects intraoperative blood pressure management during the adaptive phase of the trial and adjust if necessary. All adverse events and serious adverse events will be reported to the Medical Ethics Committee of the UMC Utrecht (see study protocol). A safety insurance policy has been taken out for all participants that covers damage as a result of medical research.

An independent study monitor (Julius Clinical) has been appointed. During on-site visits, monitoring will be conducted on enrolment progress, presence of informed consent forms for all randomised subjects, accuracy and verifiability of the recorded data from source documents, documentation of protocol deviations, safety reporting, and completeness of all other regulatory requirements.

This study will also make use of a Data Safety and Monitoring Board (DSMB). The DSMB is independent of the trial investigators and funder, and will be convened to monitor the unblended data of the trial, focusing mainly on assuring that the study follows the protocol correctly and monitors the safety issues related to the trial. The DSMB will not evaluate the efficacy of the intervention strategy. All DSMB members have signed a certification declaring no conflict of interests. The DSMB plan can be provided upon request.

## Discussion

Since the start of the PRETREAT trial, several comparable trials have been published or started elsewhere. In the POISE-3 trial, patients were randomly allocated to either a hypotension-avoidance strategy or a hypertension-avoidance strategy.^25^ In the hypotension-avoidance strategy group, the intraoperative MAP target was ≥80 mm Hg; before and for 2 days after surgery, renin–angiotensin–aldosterone system inhibitors were withheld and other long-term antihypertensive medications were administered only for systolic blood pressures ≥130 mm Hg. In the hypertension-avoidance strategy group, the intraoperative MAP target was ≥60 mm Hg; all antihypertensive medications were continued. Both intervention groups yielded similar results regarding a composite of vascular death and non-fatal myocardial injury, stroke, and cardiac arrest at 30 days. However, the intervention strategies composed multiple separate interventions from which the independent effects are not fully understood. Hence the separate parts of the strategies may lead to possible contradictory effects, which may cumulatively result in a negative trial. Also, the compliance to the administered medication was fairly low (57–75%) and the blood pressure differences between the two intervention groups were minimal. The adaptive intervention strategy of PRETREAT may result in a larger contrast between the study groups.

The currently ongoing IMPROVE multicentre, randomised trial investigates whether personalised perioperative blood pressure management reduces the incidence of the primary outcome (a composite outcome including acute kidney injury, myocardial injury, non-fatal cardiac arrest and death) within 7 days after surgery in 1272 high-risk patients undergoing elective major abdominal surgery.^26^ As described before, composite endpoints require that the frequency and severity of the individual elements included are comparable.^17^ In the case of hypotension, the incidence and severity of different organ injuries are likely not comparable. A patient-centred outcome, such as in the PRETREAT trial, captures the total impact of various organ injuries without the need for equal weights. A strength of the IMPROVE trial is the fact that blood pressure targets are individualised. However, besides the fact that it is not known if the preoperative night-time blood pressure actually represents the usual night-time blood pressure, setting the intraoperative blood pressure target at the level of the preoperative night-time blood pressure may not prevent the intraoperative blood pressure from decreasing below the determined threshold, as no safety margin is included, as is the case in the PRETREAT trial. This will likely result in more hypotension than is intended. Also, the IMPROVE trial limits the domain of the trial to high-risk abdominal surgery patients so the results of this trial will not necessarily be applicable to a broader range of patients.

Two previously published randomised controlled trials demonstrated a beneficial effect of a higher target blood pressure compared with a lower target blood pressure.^12^^,^^13^ However, in both trials the control groups seem to be undertreated compared with standard care and are thus not representative of daily clinical practice.

Obviously, the PRETREAT trial has limitations to be considered too. First, the success of the trial is dependent on the compliance of the anaesthesia team and the effect of the intervention strategy on the blood pressure. To increase the chance of successful adherence, an adaptation and implementation phase are part of the design. Because the treatment recommendations are designed by anaesthesiologists and nurse anaesthetists, the chances of a successful implementation are increased. Second, because of the intervention strategy, this is not a double-blind trial. Third, a contamination effect may result in similar outcomes for both the intervention and the control arm of the trial. However, this effect may be detected and partially mitigated by converting the design to a before–after study. Fourth, the WHODAS 2.0 questionnaire may not be sensitive enough to detect minor effects of blood pressure changes on patient outcome, especially in low-risk patients. Finally, the PRETREAT trial focuses on intraoperative hypotension and does not include the postoperative period, as adequate interventions to manage blood pressure (e.g. i.v. administration of potent vasoactive drugs) on the wards are not possible. By not intervening to avoid postoperative hypotension, it is possible that the effect on our primary outcome will be diluted.

### Research ethics approval and protocol amendments

The protocol adheres to SPIRIT guidelines, is approved by the medical ethics committee (MEC) of the UMC Utrecht (20–749), follows the Declaration of Helsinki and Good Clinical Practice, and is registered in the Dutch Trial Register (NL9391). This is protocol version 6 (22-9-2023). Future amendments will be sent to the MEC for approval.

### Confidentiality

A subject-screening and enrolment log will be kept on a secure server only accessible to study personnel. The primary and secondary endpoints will be extracted from the electronic patient record systems through the enterprise data warehouse from each centre. No extra data will be collected for this trial. All data will be pseudo-anonymised before analyses. No identifiable data will be shared between the participating hospitals. Any publication arising from this study will not contain data that can be traced back to individual patients.

### Dissemination plans

The results of this study will be communicated with others via a peer-reviewed journal. Participating patients will receive the results of this study if desired. The statistical analysis plan, patient information folder, DSMB plan and dataset will be accessible upon reasonable request after the trial is complete. No professional writers will contribute to the manuscript. Study participants will consent to having their data and results published anonymously.

## Authors’ contributions

Study concept and design: all authors

Drafting the manuscript: MK

Critical revision of the manuscript for important intellectual content: all authors

Obtained funding: TK, DV, WvK, MH

Approval of final version: all authors

## Declarations of interest

The authors declare that they have no conflicts of interest.

## Funding

The Netherlands Organization of Scientific Research (ZonMw), project ‘Rational Pharmacotherapy’ (project number: 848018005). The funding sources had no role in the study design; collection, analysis, and interpretation of data; writing of the report; or the decision to submit the report for publication.

## Appendix 1 List of collaborators

Collaborators names and academic degrees

Eline de Klerk^1^, MSc, Lisette Vernooij^2^, PhD, Luuk C. Otterspoor^3^, MD, PhD, Geert-Jan E. Cromheecke^2^, MD, Marlous Huijzer^2^, MD, Jannie Witziers^2^, BSc, Lotte E. Terwindt^1^, MD, Tim Bastiaanse^1^, BSc, Rogier V. Immink^1^, MD, PhD, Magnus Strypet^1^, MD, PhD, Niek H. Sperna Weiland^1^, MD, PhD, Marije Wijnberge^1^, MD, Marc G.H. Besselink^1^, MD, PhD, PhD, Lisette M. Vernooij^2^, PhD, Yvonne C. Janmaat^2^, MSc, Annemarie Akkermans^2^, MD, PhD

^1^Department of Anaesthesiology, Amsterdam University Medical Centre, location University of Amsterdam, Amsterdam, The Netherlands

^2^Department of Anaesthesiology, University Medical Centre Utrecht, Utrecht, The Netherlands

^3^Department of Cardiology and Department of Intensive Care Medicine, Catharina Hospital, Eindhoven, The Netherlands

## References

[1] Bijker J.B., van Klei W.A., Vergouwe Y. (2009). Intraoperative hypotension and 1-year mortality after noncardiac surgery. Anesthesiology.

[2] Hallqvist L., Granath F., Huldt E., Bell M. (2018). Intraoperative hypotension is associated with acute kidney injury in noncardiac surgery: an observational study. Eur J Anaesthesiol.

[3] Hatib F., Jian Z., Buddi S. (2018). Machine-learning algorithm to predict hypotension based on high-fidelity arterial pressure waveform analysis. Anesthesiology.

[4] Maheshwari K., Khanna S., Bajracharya G.R. (2018). A randomized trial of continuous noninvasive blood pressure monitoring during noncardiac surgery. Anesth Analg.

[5] Monk T.G., Bronsert M.R., Henderson W.G. (2015). Association between intraoperative hypotension and hypertension and 30-day postoperative mortality in noncardiac surgery. Anesthesiology.

[6] Sun L.Y., Wijeysundera D.N., Tait G.A., Beattie W.S. (2015). Association of intraoperative hypotension with acute kidney injury after elective noncardiac surgery. Anesthesiology.

[7] van Waes J.A., van Klei W.A., Wijeysundera D.N., van Wolfswinkel L., Lindsay T.F., Beattie W.S. (2016). Association between intraoperative hypotension and myocardial injury after vascular surgery. Anesthesiology.

[8] Xu L., Yu C., Jiang J. (2015). Major adverse cardiac events in elderly patients with coronary artery disease undergoing noncardiac surgery: a multicenter prospective study in China. Arch Gerontol Geriatr.

[9] Bijker J.B., van Klei W.A., Kappen T.H., van Wolfswinkel L., Moons K.G., Kalkman C.J. (2007). Incidence of intraoperative hypotension as a function of the chosen definition: literature definitions applied to a retrospective cohort using automated data collection. Anesthesiology.

[10] Salmasi V., Maheshwari K., Yang D. (2017). Relationship between intraoperative hypotension, defined by either reduction from baseline or absolute thresholds, and acute kidney and myocardial injury after noncardiac surgery: a retrospective cohort analysis. Anesthesiology.

[11] Wesselink E.M., Kappen T.H., Torn H.M., Slooter A.J.C., van Klei W.A. (2018). Intraoperative hypotension and the risk of postoperative adverse outcomes: a systematic review. Br J Anaesth.

[12] Wu X., Jiang Z., Ying J., Han Y., Chen Z. (2017). Optimal blood pressure decreases acute kidney injury after gastrointestinal surgery in elderly hypertensive patients: a randomized study: optimal blood pressure reduces acute kidney injury. J Clin Anesth.

[13] Futier E., Lefrant J.Y., Guinot P.G. (2017). Effect of individualized vs standard blood pressure management strategies on postoperative organ dysfunction among high-risk patients undergoing major surgery: a randomized clinical trial. JAMA.

[14] Chan A.W., Tetzlaff J.M., Gøtzsche P.C. (2013). SPIRIT 2013 explanation and elaboration: guidance for protocols of clinical trials. BMJ.

[15] Wesselink E.M., Wagemakers S.H., van Waes J.A.R., Wanderer J.P., van Klei W.A., Kappen T.H. (2022). Associations between intraoperative hypotension, duration of surgery and postoperative myocardial injury after noncardiac surgery: a retrospective single-centre cohort study. Br J Anaesth.

[16] Ustün T.B., Chatterji S., Kostanjsek N. (2010). Developing the World Health Organization disability assessment schedule 2.0. Bull World Health Organ.

[17] Freemantle N., Calvert M., Wood J., Eastaugh J., Griffin C. (2003). Composite outcomes in randomized trials: greater precision but with greater uncertainty?. JAMA.

[18] Shulman M.A., Kasza J., Myles P.S. (2020). Defining the minimal clinically important difference and patient-acceptable symptom state score for disability assessment in surgical patients. Anesthesiology.

[19] Moonesinghe S.R., Jackson A.I.R., Boney O. (2019). Systematic review and consensus definitions for the Standardised Endpoints in Perioperative Medicine initiative: patient-centred outcomes. Br J Anaesth.

[20] Meng L. (2021). Heterogeneous impact of hypotension on organ perfusion and outcomes: a narrative review. Br J Anaesth.

[21] Ackland G.L., Abbott T.E.F. (2022). Hypotension as a marker or mediator of perioperative organ injury: a narrative review. Br J Anaesth.

[22] R-Core-Development-Team (2019).

[23] Kappen T.H., Moons K.G., van Wolfswinkel L., Kalkman C.J., Vergouwe Y., van Klei W.A. (2014). Impact of risk assessments on prophylactic antiemetic prescription and the incidence of postoperative nausea and vomiting: a cluster-randomized trial. Anesthesiology.

[24] Kappen T.H., van Klei W.A., van Wolfswinkel L., Kalkman C.J., Vergouwe Y., Moons K.G.M. (2018). Evaluating the impact of prediction models: lessons learned, challenges, and recommendations. Diagn Progn Res.

[25] Marcucci M., Painter T.W., Conen D. (2023). Hypotension-avoidance versus hypertension-avoidance strategies in noncardiac surgery. Ann Intern Med.

[26] Bergholz A., Meidert A.S., Flick M. (2022). Effect of personalized perioperative blood pressure management on postoperative complications and mortality in high-risk patients having major abdominal surgery: protocol for a multicenter randomized trial (IMPROVE-multi). Trials.

