# Supplementary material for: The effect of proactive versus reactive treatment of hypotension on postoperative disability and outcome in surgical patients under anaesthesia (PRETREAT): clinical trial protocol and considerations
Source: BJA Open. 2024 Feb 29;9:100262. doi: 10.1016/j.bjao.2024.100262 (PMC10910055; doi:10.1016/j.bjao.2024.100262)

PRETREAT trial

The effect of **p**roactive versus **re**active **treat**ment of hypotension on postoperative disability and outcome in surgical patients under anaesthesia (PRETREAT): an adaptive, multicentre randomized controlled trial

September 2023

Department of Anaesthesiology, University Medical Centre Utrecht, Utrecht, the Netherlands
M. Kant MD, T.H. Kappen MD PhD, W.A. van Klei MD PhD

Department of Anaesthesiology, Amsterdam University Medical Centre, Amsterdam, the Netherlands
D. P. Veelo MD PhD, M.W. Hollmann MD PhD

PROTOCOL TITLE: The effect of proactive versus reactive treatment of hypotension on postoperative disability and outcome in surgical patients under anaesthesia (PRETREAT): an adaptive, multicentre randomized controlled trial

**Protocol ID**	NL72175.041.20
**Short title**	PRETREAT
**Version**	Version 6
**Date**	22-9-2023
**Sponsor**	UMC Utrecht
**Coordinating investigator/project leader**	UMC Utrecht:<br>Dr. T.H. Kappen<br>E-mail: T.Kappen@umcutrecht.nl
**Principal investigators**	UMC Utrecht:<br>Dr. T.H. Kappen<br>E-mail: T.Kappen@umcutrecht.nl<br><br>University Medical Centre Amsterdam, location AMC:<br>Dr. D.P. Veelo<br>E-mail: d.p.veelo@amsterdamumc.nl
**Contact**	UMC Utrecht<br>Department of Anaesthesiology<br>3508 GA Utrecht<br>E-mail: m.kant-2@umcutrecht.nl<br>Telephone: +31 (0)6 466 448 22
**Independent experts**	UMC Utrecht:<br>Dr. Marije Marsman<br>E-mail: M.Marsman-2@umcutrecht.nl<br>Telephone: 0887574388<br><br>University Medical Centre Amsterdam, location AMC:<br>Prof. Dr. Benedikt Preckel<br>E-mail: b.preckel@amsterdamumc.nl<br>Telephone: +31 20 5669111

**Subsidising party**	Zon MW Goed Gebruik Geneesmiddelen<br>Project nummer 848018005

PROTOCOL SIGNATURE SHEET

**Name**	**Signature**	**Date**
**Sponsor or legal representative:**<br><br>Prof. Dr. A.M.G.A. de Smet<br>*Medisch wetenschappelijk manager*		
**Principal Investigator:**<br><br>T.H. Kappen		

TABLE OF CONTENTS

1. INTRODUCTION AND RATIONALE 11
1.1 Background: current anesthesia practice 12
2. OBJECTIVES 14
3. STUDY DESIGN 15
4. STUDY POPULATION 16
4.1 Population (base) 16
4.2 Inclusion criteria 16
4.3 Exclusion criteria 16
4.4 Sample size calculation 16
5. TREATMENT OF SUBJECTS 19
5.1.1 Component 1 – Target blood pressure 19
5.1.2 Component 2 – The guidelines to achieve target blood pressure 20
5.1.3 Care-as-usual 21
5.1.4 Adaptation cycles 21
6. METHODS 23
6.1 Main study parameter/endpoint 23
6.2 Secondary study parameters/endpoints 23
6.3 Randomisation, blinding and treatment allocation 25
6.4 Study procedures 25
6.5 Withdrawal of individual subjects 27
6.6 Replacement of individual subjects after withdrawal 27
6.7 Follow-up of subjects withdrawn from treatment 28
6.8 Premature termination of the study 28
6.9 The impact of COVID-19 on this study 28
6.10 Protocol deviation and violation 29
7. SAFETY REPORTING 30
7.1 Temporary halt for reasons of subject safety 30
7.2 AEs, SAEs 30
7.2.1 Adverse events (AEs) 30
7.2.2 Serious adverse events (SAEs) 30
7.3 Follow-up of adverse events 31
7.4 Data Safety Monitoring Board (DSMB) / Safety Committee 31
8. STATISTICAL ANALYSIS 32
8.1 Primary study parameter 33
8.2 Secondary study parameters 33
8.3 Other study parameters 34
8.4 Interim analysis 34
9. ETHICAL CONSIDERATIONS 36
9.1 Regulation statement 36
9.2 Recruitment and consent 36
9.3 Benefits and risks assessment, group relatedness 36

9.4 Compensation for injury 38
9.5 Incentives 38
10. ADMINISTRATIVE ASPECTS, MONITORING AND PUBLICATION 39
10.1 Handling and storage of data and documents 39
10.2 Monitoring and Quality Assurance 39
10.3 Amendments 39
10.4 Annual progress report 39
10.5 Temporary halt and (prematurely) end of study report 40
10.6 Public disclosure and publication policy 40
11. REFERENCES 41
12. APPENDICES 44
12.1 Appendix 1: risk score to determine target blood pressure 44
12.2 Appendix 2 Guidelines to achieve target blood pressure 46
12.3 Appendix 3 Overview of the different study phases 47

LIST OF ABBREVIATIONS AND RELEVANT DEFINITIONS

**ABR**	**General Assessment and Registration form (ABR form), the application form that is required for submission to the accredited Ethics Committee; in Dutch: Algemeen Beoordelings- en Registratieformulier (ABR-formulier)**
**AE**	**Adverse Event**
**ASA**	**American Association of Anaesthesiologists**
**AMC**	**Academic Medical Centre**
**CCMO**	**Central Committee on Research Involving Human Subjects; in Dutch: Centrale Commissie Mensgebonden Onderzoek**
**CV**	**Curriculum Vitae**
**DSMB**	**Data Safety Monitoring Board**
**EU**	**European Union**
**EQ-5D-5L**	**5 level EQ-5D version by EuroQol**
**GCP**	**Good Clinical Practice**
**GDPR**	**General Data Protection Regulation; in Dutch: Algemene Verordening Gegevensbescherming (AVG)**
**IC**	**Informed Consent**
**IOH**	**Intraoperative hypotension**
**MAP**	**Mean Arterial Pressure**
**METC**	**Medical research ethics committee (MREC); in Dutch: medisch-ethische toetsingscommissie (METC)**
**(S)AE**	**(Serious) Adverse Event**
**Sponsor**	**The sponsor is the party that commissions the organisation or performance of the research, for example a pharmaceutical company, academic hospital, scientific organisation or investigator. A party that provides funding for a study but does not commission it is not regarded as the sponsor, but referred to as a subsidising party.**
**UAVG**	**Dutch Act on Implementation of the General Data Protection Regulation; in Dutch: Uitvoeringswet AVG**
**UMCU**	**University Medical Centre Utrecht**
**WHODAS**	**World Health Organisation Disability Assessment Schedule**
**WMO**	**Medical Research Involving Human Subjects Act; in Dutch: Wet Medisch-wetenschappelijk Onderzoek met Mensen**

SUMMARY

Rationale: Over 1.4 million surgical procedures are performed every year in the Netherlands, of which one million under general or regional anaesthesia.(1) Unfortunately, surgery is not without complications. A risk factor for complications that commonly occurs during surgery under anaesthesia is low blood pressure (hypotension). Anaesthesiologists have been using cardiovascular drugs since the emergence of large-scale anaesthesia to treat hypotension, but despite those efforts, in more than 75% of surgical procedures (approximately 750.000 procedures per year) patients have one or more episodes of hypotension (a Mean Arterial Pressure (MAP) below 65 mmHg).(2–4) Patients with more and greater blood pressure fluctuations have a greater chance of their blood pressure dropping below the currently advocated minimal acceptable threshold of a MAP of 65 mmHg.

A stricter blood pressure management to prevent hypotension in a larger population is not straightforward. Applying the right drug in an appropriate amount at an appropriate time is surprisingly challenging.

The current paradigm of blood pressure management is predominantly reactive: blood pressure is treated when it approaches a specified minimally acceptable threshold (for example MAP 65) or when it is rapidly dropping (5). From a risk perspective it makes more sense to shift the paradigm to a proactive approach: setting higher blood pressure thresholds so that the anaesthesiologist will intervene earlier to keep the blood pressure at a certain margin above the specified blood pressure threshold. Patients at greater risk of severe blood pressure fluctuations need to be kept at a higher target blood pressure to keep them above the threshold. This requires a strategy that allows the anaesthesia team to keep their patients' blood pressures at the appropriate level.

Objective: The aim of this adaptive multicentre randomized controlled trial is to maintain patients at a target blood pressure level with a sufficient margin from a minimal acceptable blood pressure threshold of a MAP of 65 mmHg to reduce the incidence of hypotension. We will investigate whether a proactive blood pressure management approach improves functional disability at six months compared to the reactive blood pressure management approach, i.e. care as usual, in adult patients after elective noncardiac surgery.

Study design: A multicentre adaptive randomized controlled trial. Patients will be either randomized to the intervention (proactive blood pressure management strategy) or care-as-usual (predominantly reactive blood pressure management strategy).The proactive blood pressure management strategy will be evaluated and further revised in adaptation cycles of three weeks. At three and six months into the trial, the need for continuing the adaptation cycles will be reflected. When the intervention has a successful impact on the clinical process and no or little further progress is expected, the adaptation

cycles will end and only the evaluation of the effect of the treatment strategies will continue.

Study population: Adult patients scheduled for elective non-cardiac surgery under general anaesthesia or central neuraxial anaesthesia with a scheduled postoperative hospital stay of at least one night– i.e. inpatients – will be considered eligible for inclusion.

Intervention: The intervention is the proactive risk based blood pressure management strategy that keeps the blood pressure at a set margin and avoids dropping below the minimal acceptable threshold of a MAP of 65 mmHg.(2,4) The proactive strategy consists of two components: 1) a target blood pressure that provides a sufficient margin, based on the individual risk on developing hypotension; and 2) a clinical guideline with suggestions how to keep patients at their target blood pressure.
Patients with a high likelihood of intraoperative hypotension (IOH; MAP < 65 mmHg) should require larger margins and thus higher target blood pressures compared to those with a low likelihood to develop IOH. Therefore patients will be divided into low-, intermediate- and high-risk groups based on their IOH likelihood, with resulting blood pressure targets of MAP 70, 80, and 90 mmHg respectively.

Main study parameters/endpoints:
The primary outcome of the study is functional disability at six months after surgery, measured with the 12-item World Health Organization Disability Assessment Score (WHODAS) 2.0, which reflects difficulties experienced in different functional domains (cognition, mobility, self-care, getting along, life activities and participation in the previous 30 days).(6–8)
Three levels of secondary outcomes can be distinguished: 1) at the level of changes in the behavior of the anaesthesia team; 2) the change in blood pressures and prevention of hypotension; and 3) the impact on patient outcome. The behavioral impact is measured through changes in blood pressure interventions, e.g. dosages of cardiovascular drugs, time to first intervention after a blood pressure dropped below a particular threshold. At the blood pressure level, the incidences, depths and durations of both intraoperative hypotension and hypertension will be evaluated. At the patient outcome level, disability (WHODAS 2.0) and quality of life (EQ-5D-5L)(9) at 30 days postoperatively, quality of life measured (EQ-5D-5L) 6 months postoperatively, and all-cause mortality within 6 months will be studied. In addition, short term effects – i.e. the effects on a patient's hospital – will be evaluated, e.g. in-hospital mortality, the incidence of several complications, length of hospital stay and estimated intraoperative blood loss. For a complete list of endpoints we refer to sections 6.1 and 6.2.

Nature and extent of the burden and risks associated with participation, benefit and group relatedness:

Benefit:

With the proactive risk-based intervention strategy, a low blood pressure may be avoided. Avoiding intraoperative low blood pressure might result in less functional disability after surgery. Besides the possible effect of this intervention strategy for the patient, it is likely that avoiding hypotension and possibly reducing complications after surgery will reduce the healthcare costs by millions of euros.

Burden:

The burden for participating patients is considered low. All interventions will take place whilst the patient is undergoing surgery. Patients need to complete two questionnaires at three time points which are part of standard of care. Patients do not need to perform any additional actions or undergo additional procedures.

Risks:

The risk of an intra-operative higher blood pressure is low. This study will ask the anaesthesia team to intervene to prevent a patients' blood pressure from dropping and/or treat the patient when hypotensive. The anaesthesia team remains fully responsible for the chosen treatment and whether the strategy is feasible for an individual patient. The anaesthesia team will likely maintain or reduce the dosages of cardiovascular drugs when the blood pressure is adequate or too high (i.e. above nadir blood pressures before surgery). This reduction of the dosages are left at the discretion of the anaesthesia teams as this already an intricate part of their job standing. Thus, this negates the risk of severe intraoperative hypertension. However, complications due to the intervention strategy cannot be completely excluded. Consequently, the risk of participating in this study is moderate.

1. INTRODUCTION AND RATIONALE

Over 1.4 million surgical procedures are performed every year in the Netherlands, of which one million under general or regional anaesthesia.(1) Unfortunately, surgery is not without complications. When a patient undergoes anaesthesia, the body loses control over the compensatory mechanisms to regulate blood pressure. This commonly results in hypotension. Hypotension, even for one minute, has been reported to be associated with complications, such as myocardial infarction, renal failure, and postoperative death.(2,10) When complications like these become persistent they likely lead to persistent functional disability. Whether or not hypotension is related to complications and whether or not preventing hypotension results in less complications still has to be elucidated. Therefore, unraveling these mechanisms has been prioritized in the 2018 TOP-10 research agenda of the Dutch Society of Anaesthesiology.(11)

Anaesthesiologists have been using cardiovascular drugs since the emergence of large-scale anaesthesia to counterbalance hypotension, but despite those efforts, in more than 75% of surgical procedures (approximately 750.000 procedures per year) patients have one or more episodes of hypotension (a Mean Arterial Pressure (MAP) below 65 mmHg).(2–4)

Anaesthetic drugs, surgical manipulation and blood loss typically cause many fluctuations in a patient’s blood pressure. Not all patients are affected equally: the frequency and magnitude of blood pressure fluctuations depend on the type of surgery and on the patient’s comorbidity. Patients with more and greater blood pressure fluctuations have a greater chance of their blood pressure dropping below the currently advocated minimal acceptable threshold of a MAP of 65 mmHg.

A stricter blood pressure management to prevent hypotension in a larger population is not straightforward. Applying the right drug in an appropriate amount at an appropriate time is surprisingly challenging. A recent study suggests that a higher target blood pressure – well above MAP 65 mmHg – may improve patient outcome.(12) Nonetheless, the intervention strategy in that study was aimed at a very high-risk group of patients with a specific treatment protocol, which cannot easily be adopted for use in the larger surgical patient population. This is similar to earlier research on improving hemodynamics through the use of advanced monitoring.(13) Advanced monitoring typically involves costly new devices that require highly specialist knowledge and training. Hence, wide-scale adaptation of these complex techniques has not become commonplace, nor is it expected to be in the near future.

Other recent efforts have tried more broadly applicable interventions to prevent hypotension by providing automated alerts of low blood pressures to the anaesthesia team, i.e. clinical decision support.(14,15) Unfortunately, without any clinically relevant success. It may seem obvious that increased focus on avoiding low blood pressures should result in less hypotension. After all, blood pressure management is a major focus of the anaesthesia team. However, the alerts were only

informative (assistive) and did not include an advice (directive). Assistive alerts do not provide information about the appropriate course of action. When the appropriate course of action is not obvious, assistive alerts may not prompt users to action, i.e. the alerts are not actionable.(16,17) We thus need an actionable strategy for blood pressure management that is different from the current paradigm of blood pressure management.

The current paradigm of blood pressure management is predominantly reactive: blood pressure is treated when it approaches the minimally acceptable threshold (for example MAP 65) or when it is rapidly dropping. From a risk perspective it makes more sense to shift the paradigm to a proactive approach: setting higher blood pressure thresholds so that the anaesthesiologist will interference earlier to keep the blood pressure at a set margin above the possibly dangerous blood pressure threshold. Patients at greater risk of severe blood pressure fluctuations need to be kept at a higher target blood pressure. This requires a strategy that allows the anaesthesia team to keep their patients' blood pressures at the appropriate level.

1.1 Background: current anesthesia practice

The anesthesia team uses high dose anesthetics and analgesics to not only keep patients unaware of the procedure and free from pain, but also to keep the patient's immediate stress response under control. The anesthetics and analgesics have major respiratory and hemodynamic consequences that need to be treated, e.g. the anesthesia team takes over respiration through intubation and mechanical ventilation.

The hemodynamic consequences are a bit more complicated: anesthetics and analgesics modify sympathetic tone, cause vasodilation in both the arterial and venous vascular systems, and decrease myocardial function. The loss of sympathetic tone and vasodilation have the largest impact on blood pressure and blood flow, resulting in intraoperative hypotension. Hence, the main hemodynamic intervention of the anesthesia team is to administer vasoactive drugs to negate the vasodilation and maintain an adequate blood pressure. Other interventions include intravenous fluid administration, adjusting maintenance dosages of anesthetics and analgesics, and administration of other cardiovascular drugs, such as inotropes and chronotropes. However, under regular anesthesia conditions during elective surgical procedures, the effects of the other interventions are limited as they do not address the main cause of intraoperative hypotension.

The situation of anesthesia-induced hypotension and hypotension caused by the physiological changes induced by body position and the surgery itself is markedly different from other causes of intraoperative hypotension. In such situations, intraoperative hypotension is typically caused by specific unforseen events (major hemorrhage, accidental vena cava compression) – but can also be the development of unforeseen medical conditions (e.g. electrolyte disturbances, arrythmias, or

anaphylaxis). In addition, it can also be part of the patient’s primary diagnosis in emergency conditions, such as patients with major trauma or septic shock. In contrast to anesthesia-and surgery induced hypotension, the hemodynamic changes caused by such events are typically more sudden and can be much more profound, even resulting in circulatory shock.

When studying the question ‘At what blood pressure levels should patients be kept during surgery?’, we thus have to differentiate between expected fysiological changes due to the procedure and anesthesia-induced hypotension and on the other hand the more unpredictable and situation-dependent hypotension induced by specific events. The former is very common and treatment is easier to standardize. The latter, event-induced hypotension is very difficult to standardize and needs a tailored solution.

In clinical practice, the distinction between anesthesia-induced hypotension and event-induced hypotension is not always straightforward. Consequently, the research question can only be studied through a pragmatic study design. The anesthesia team is encouraged to use the proactive treatment strategy to prevent the more mild, anesthesia-induced hypotension or expected hypotension due to physiological changes caused by the procedure, while the individual treatment decisions on blood pressure management remain at the discretion of the anesthesiologist to ensure appropriate treatment of event-induced hypotension.

2. OBJECTIVES

The aim of this adaptive multicentre randomized controlled trial is to maintain patients at a target blood pressure level with a sufficient margin from the minimal acceptable blood pressure threshold of a MAP of 65 mmHg. This study in adult patients undergoing elective non-cardiac surgery will investigate whether a proactive blood pressure management approach reduces the incidence of hypotension and improves functional disability at six months compared to the reactive blood pressure management approach, i.e. care as usual.

3. STUDY DESIGN

This study is a multicentre, adaptive randomized controlled trial. A risk-based intervention strategy will be implemented, further refined (see 5.4 adaptation cycles) and studied for its impact in an adaptive randomized controlled trial. Patients will be divided into three risk strata: a low, intermediate or high risk of intraoperative hypotension, according to their calculated risk scores (see appendix 1). Subsequently, patients will be randomized to either the intervention (proactive risk-based intervention strategy) or care-as-usual (predominantly reactive). Patients are either treated at the University Medical Centre Utrecht (UMCU) or at one of the locations of the Amsterdam University Medical Centre. The different phases of the trial are displayed in Appendix 3.

4. STUDY POPULATION

4.1 Population (base)

Adult patients scheduled for elective non-cardiac surgery under general anaesthesia or central neuraxial anaesthesia at one of the participating centres, with a scheduled postoperative hospital stay of at least one night will be considered eligible for inclusion.

4.2 Inclusion criteria

In order to be eligible to participate in this study, a subject must meet all of the following criteria:

- Adult ≥18 years or older
- Elective, non-cardiac surgery under general anaesthesia or central neuraxial anaesthesia
- Expected hospital stay of at least one night after surgery

4.3 Exclusion criteria

A potential subject who meets any of the following criteria will be excluded from participation in this study:

- Low risk surgery: Opthalmic surgery, endoscopic gastrointestinal procedures, (interventional) radiologic procedures, obstetric procedure
- Organ transplantation
- Procedures with a scheduled surgical time of less than 30 minutes
- Participation in another clinical trial that is interfering with the procedures and outcomes of the PRETREAT trial
- Patients unable to fully comply to study needs (e.g. legally incapable patients or patients unable to communicate in Dutch or English).
- Patients with an American Society of Anaesthesiologists (ASA) Physical status 5

4.4 Sample size calculation

Prior to the start of this study a sample size analysis was performed based on the literature which yielded a sample size of 2,500 patients in the intermediate-risk stratum with an overall sample size of 5,000 patients divided over two years and two centres.

To update the sample size analysis we collected WHODAS 2.0 questionnaires from patients who underwent surgery in both the UMC Utrecht and Amsterdam UMC, location AMC. A total of 770 questionnaires were collected (UMC Utrecht n=700, Amsterdam UMC, location AMC n=70) before the collection was interrupted due to the COVID-19 crisis. The mean and standard deviation of the available questionnaires were used to recalculate the required sample size.

In our initial sample size analysis we used an expected 12%-point decrease in mean WHODAS at six months with a standard deviation of 32%-point for the highest IOH risk stratum, which was based on recent literature.(18,19) As we expect the low-risk group to have a negligible effect size, we estimated the effect size of the intermediate-risk group at halfway the low-risk and high-risk group, that is a 6%-point change in functional disability with a standard deviation of 23%-point.
However, as the overall mean and standard deviation of the WHODAS 2.0 score were lower in the UMC Utrecht and Amsterdam UMC than in those previous studies, we adjusted the expected effect sizes and variances accordingly and recalculated our estimated sample size. The updated sample size was calculated to detect a 5%-point change (from 17% to 12%) in functional disability at six months in the intermediate-risk stratum with a 17%-point within-cluster standard deviation, and a between-cluster standard deviation of 0.5%-point. The sample size was increased by 20% to account for possible loss-to-follow-up. We calculated a cluster-adjusted sample size using the clusterPower package (version 0.6.111) in R software.(20) This resulted in an estimated sample size of 2,248 patients in the intermediate-risk stratum, with an overall sample size of 4,496 patients divided over two study groups at two centres over two years inclusion.

Due to the COVID-19 crisis only a limited amount of questionnaires were available to calculate the sample size and power (n=770). Hence, true means and standard deviations of the WHODAS 2.0 scores within our populations might differ slightly from this sample. Because of this uncertainty and that the updated sample size is close to the original estimated sample size based on literature, we decided to stay with the original estimated sample size of 5,000 patients and performed a power analysis with that sample size (see Table 1). This results in 0.94 power to detect an overall difference of 5%-point (from 17% to 12%) in functional disability as measured by the WHODAS 2.0 questionnaire.

Both centres perform approximately 2,500 eligible procedures per year for elective non-cardiac surgery of intermediate to high risk. A 50% rate of informed consent would still be sufficient to reach the total sample size of 5,000 patients in two years in two centres. Both participating centres have declared that the inclusion rate is expected to be feasible.

Table 1 – Sample size and power calculations

Risk stratum	Est. effect size	Within-cluster SD	Between-cluster difference in effect size	CV	Power	Alpha	n_{total}	$n_{cluster}$
Low			No significant result expected				1250	625
Intermediate	5%	17	0.5%	0.8	0.83	0.05	2500	1250
High	10%	24	1%	0.8	0.83	0.05	1250	625

**Total**	**5%**	**17**	**0.5%**	**0.8**	**0.94**	**0.05**	**5000**	**2500**

SD = standard deviation; CV = coefficient of variation for differences in cluster sizes

Sample sizes are presented after a 1.2 correction for possible loss-to-follow-up, whereas power is calculated without that correction.

5. TREATMENT OF SUBJECTS

The hypothesis of this study is that a proactive strategy keeps the blood pressure at a sufficient margin and avoids dropping below the minimal acceptable threshold of a MAP of 65 mmHg.(2,4) The proactive strategy consists of two components: 1) a target blood pressure that provides a sufficient margin; 2) a clinical guideline with suggestions to keep patients at their target blood pressure.

5.1.1 Component 1 – Target blood pressure

In a reactive strategy, the anaesthesia team typically aims for a target blood pressure that is the same or close to the minimal acceptable blood pressure threshold. Consequently, unanticipated decreases in blood pressure quickly result in a patient's blood pressure crossing the minimum acceptable threshold (a MAP of 65 mmHg), which possibly increases the risk of organ injury.(5) In contrast, a proactive strategy would aim for a target blood pressure that includes enough margin to keep patients away from the minimal acceptable threshold, even when unanticipated decreases in blood pressure occur (see *Figure 1*).

Figure 1. Intervention and hypothesis rationale

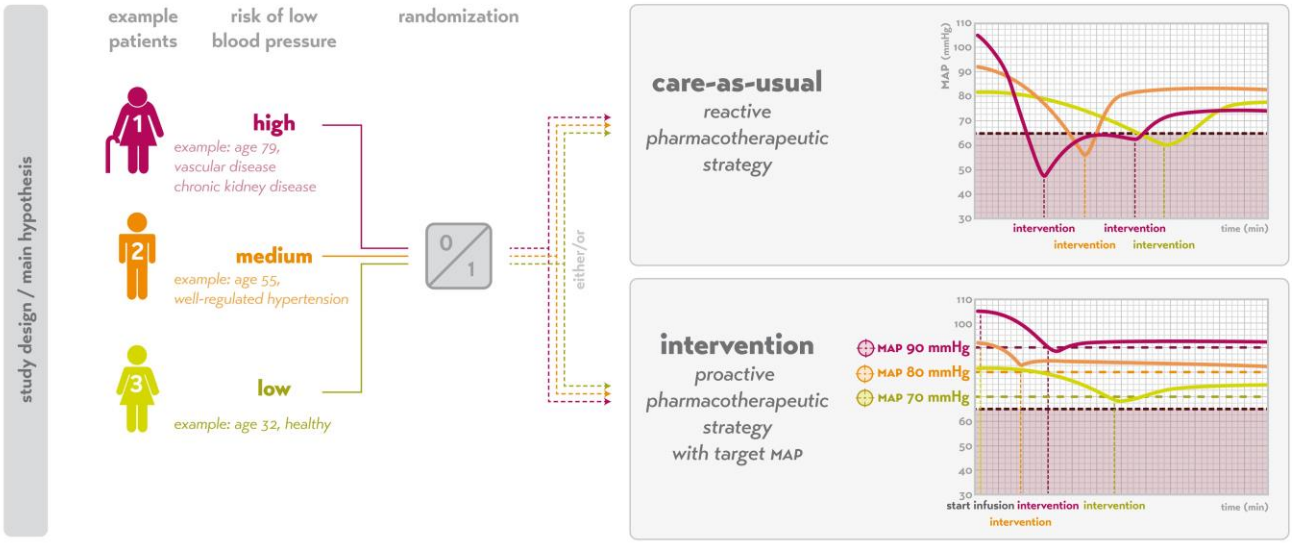

Patients with a high likelihood of IOH require larger margins and thus higher target blood pressures compared to those with a low IOH likelihood. In the current literature, no comprehensive list of risk factors is available for this purpose. Using a multivariable regression approach on combined historic data from the UMC Utrecht and the Amsterdam AMC, a risk score for severe/prolonged IOH was developed. Based on their hypotension risk score – i.e. their individual likelihood of IOH – patients will be divided into low-, intermediate- and high-risk strata, with resulting blood pressure targets of MAP 70, 80, and 90 mmHg respectively. The low-risk stratum will include the quartile of patients with the lowest IOH likelihood, the intermediate-risk stratum will include the middle two quartiles (i.e. the

interquartile range), and the high-risk stratum will include the quartile of patients with the highest likelihood of IOH (see Appendix 1).

The IOH threshold for organ injury may be different between individual patients. Although we could speculate which groups are more susceptible (e.g. pre-existent hypertension, patients undergoing carotid surgery), we do not know which individual needs which target blood pressure. Hence, anaesthesiologists may change the target blood pressure when they deem it necessary for specific patients, which will be documented in the anaesthesia information management system.

5.1.2 Component 2 – The guidelines to achieve target blood pressure

Setting a target blood pressure does not automatically reduce the incidence of IOH. To keep patients at those higher blood pressure targets, a more liberal guideline to achieve the target blood pressure than our current clinical practice is required. Our hypothesis is that current clinical practice falls short in: a) the anticipated treatment of the vasodilatory side effects of anaesthesia; b) the response to unanticipated decreases in blood pressure.

For this study, we developed a clinical guideline (see Appendix 2) that can be used to achieve the target blood pressure for patients in the intervention arm of this study. The clinical guideline provides how to proactively deploy treatment options that currently being used in clinical practice to treat intraoperative hypotension. Different centres use different vasopressor drugs: at the UMC Utrecht phenylephrine often is a first choice for the continuous infusion of vasopressors, whereas the Amsterdam UMC primarily uses norepinephrine. Both centres also use ephedrine in intermittent boluses to increase blood pressure. As no convincing evidence exists that any single drug improves outcome over the others, each centre will keep using their preferred vasopressors. The guidelines developed for this study have the same core components for both centres, so each centre will use the same class of drugs in similar clinical situations, i.e. when the cause of hypotension is the same. The actual drug used may differ between centres and hence the dosing regimen will be adapted to local practices. This way our proactive strategy will have the highest chance of success of widespread implementation in the Netherlands and abroad.

The clinical guidelines are summarized into a ‘reinforcement flowchart’ suggesting when to treat and what and will be available both on paper and as a reference link within the anaesthesia information management systems. The aim of the intervention strategy is to maintain patients at a higher target blood pressure, not to instruct the anesthesiologist to make specific treatment decisions to reach the target blood pressure. The clinical guideline only provides suggestions and is not considered a study treatment protocol with strict adherence. The attending anaesthesiologist can make patient-specific adjustments to the intervention strategy. Such adjustments include adjusting the target blood pressure or use a different vasopressor dosing regimen than suggested in the clinical guideline. Interventions are already documented in the electronic patient record, and the anaesthesia

team will further be encouraged to document reasons for making patient-specific adjustments.

The initial clinical guideline was developed by a group of clinical champions from each center together with the research team. The clinical champions team consisted of an anesthesiologist, a nurse anesthesist and an anesthesiology resident. They were instructed to design a guideline for proactive blood pressure management that is effective, feasible and safe. For more information regarding the safety and risks of the intervention strategy we refer to section 9.3.

5.1.3 Care-as-usual

As blood pressure management is an intricate part of routine anaesthesia management, it would be unethical to keep the control group patients untreated for low blood pressure. A protocol that would allow only treatment when the blood pressure is already below the minimally acceptable blood pressure threshold (a MAP < 65 mmHg) would also be problematic and not represent our current clinical practice. The target blood pressure for control group patients will not be set by protocol (care-as-usual).

5.1.4 Adaptation cycles

The first six months of the trial, the risk-based interventions strategy will be evaluated and further revised in adaption cycles of three weeks (see Appendix 3). The aim of the adaption cycles is to improve the intervention strategy, further empowering the team members to keep patients at their target blood pressure. In addition, the intervention strategy will be adjusted to avoid intraoperative high blood pressure (hypertension).

The impact of the interventions will be quantified by the depth and duration of hypotension, and cardiovascular drug use (dose, timing, type). Data extraction and data processing are done by an automated script, which will allow us to perform the analysis triweekly. In addition, weekly interviews with members of the anaesthesia staff and faculty will provide in-depth clinical insights, as well as facilitators and barriers for implementation. Based on the information from the quantitative and qualitative analysis, changes can be made to improve the intervention strategy (examples of changes that can be made to the intervention strategy are provided in section 8.4). Adjustments will be made by the research team members and presented to the clinical champions of both hospitals every three weeks. Once all research team members and clinical champions agree with the adjustments the adjusted intervention strategy will be evaluated for the next three weeks in both hospitals.

At three and six months into the trial, the need for continuing the adaption cycles will be reflected. The intervention is considered successful if it results in a 30% or more relative risk reduction of intraoperative hypotension. Also the clinical champions should agree that the intervention strategy is easy to implement in practice and the reduction in hypotension should be accompanied by a clear change in intraoperative administration of cardiovascular drugs (e.g. timing, dose, type). The results

are discussed in a meeting after three and six months with all the research team members and the clinical champions. When the intervention has a successful impact on the clinical process and no or little further progress is expected, the adaption cycles will end and only the evaluation of the effect of the treatment strategies will continue during meetings every six weeks. The aim is not to make substantial changes to the intervention strategy after the adaption phase. However, changes can be made to the intervention strategy if considered necessary, for example when compliance with the guidelines proves to be difficult in certain uncommon clinical situations that may not have occurred yet during the adaptation phase (e.g. induction with etomidate that may require a starting dose adjustment). For more details about the adaptation cycles see section 8.4 ‘Interim Analysis’.

6. METHODS

6.1 Main study parameter/endpoint

The primary outcome is disability at six months after surgery, measured by the 12-item World Health Organization Disability Assessment Score (WHODAS) 2.0 scale.(6,8,21) The WHODAS scale records difficulties experienced in different functional domains including, cognition, mobility, self-care, getting along, life activities and participation during the previous 30 days. Disability is defined as a decrement in each functioning domain corresponding to score between 0% and 100%, in which no disability stands for a score of 0% and full disability represents a score of 100%, including death. The WHODAS 2.0 is easy to use and patient centered. In a validation study including non-cardiac surgical patients, the WHODAS 2.0 has been found to be a clinically acceptable, valid, reliable and responsive instrument for measuring postoperative disability.11,12 Moreover, as multiple organ systems are susceptible to low organ perfusion, the primary endpoint of the study should reflect the variation in possible adverse effects of IOH, not only a specific organ injury due to possible hypoperfusion. Hence, the primary endpoint is measured with the 12-item WHODAS 2.0 scale. This is accordance with the recommendation of the Standardized Endpoints for Perioperative Medicine initiative (STEP) published by the British Journal of Anaesthesia (22).

6.2 Secondary study parameters/endpoints

Endpoints: patient level

- Disability at 30 days after surgery, measured with the 12-item WHODAS 2.0
- Quality of life at 30 days and 6 months postoperatively, measured with the EuroQoL 5D-5L questionnaire(9)
- Clavien-Dindo classification and surgical complications as registered within the DICA database (23, 24)
- In-hospital mortality within 48 hours and during hospital stay
- All-cause mortality within 6 months
- Length of hospital stay
- Troponin in first week after surgery when available from routine clinical care
- Creatinine in first week after surgery when available from routine clinical care
- Intensive Care admission during hospital stay
- Non-prophylactic antibiotics during hospital stay in first week after surgery
- Readmission at the same hospital within 6 months after surgery
- Reoperation during hospital stay
- Hemorrhage requiring blood transfusion after surgery
- Onset of cardiac complications 24 hours after surgery

    - New-onset atrial fibrillation
    - New-onset heart failure and/or ischemia
- In-hospital life-threatening events (as reported by diagnosis code in electronic health record)
    - Shock
    - Deep vein thrombosis or pulmonary embolism
    - Myocardial infarction
    - Cerebral vascular accident
    - Acute kidney injury

Endpoints: process level

- The incidence of intraoperative hypotension
- The depth and duration of intraoperative hypotension
- Estimated intraoperative blood loss in ml

Endpoints: behavioral level

- Intraoperative anaesthetic/analgesic drug use
- average intraoperative use of the following blood pressure interventions
    - Vasopressor use
    - Inotropic use
    - Intravenous fluid administration
    - Blood products administration
- Time to first blood pressure intervention after MAP drops below target blood pressure at any point during the anaesthetic case

Determinants

- Disability before surgery, measured with the 12-item WHODAS 2.0
- Quality of life before surgery, measured with the EuroQoL 5D-5L questionnaire
- Gender
- Age
- Surgery specialty
- Duration of procedure
    - Planned duration
    - Actual duration
- ASA-class
- Comorbidity

    - Hypertension
    - Cardiac diseases
    - Diabetes
- Type of anesthesia
    - General anesthesia
    - Central neuraxial anesthesia

<u>Data collection</u>

All required data are routinely collected within the current standards of care. The data will be extracted from the electronic patient record systems through the enterprise data warehouse from each centre. During the adaption cycles of the trial, all determinants, process level and behavioural level parameters will be extracted from the electronic patient record system every three weeks. For the full duration of the trial, we will extract data on cardiology consultation and/or ECG examination within 24 hours after surgery, and in-hospital mortality from the electronic patient record system every three weeks. We will review the medical charts of patients who received cardiology consultation and/or ECG examination to detect new onset atrial fibrillation and/or new onset heart failure and/or ischemia. Every year all other patient level parameters will be extracted from the electronic patient record system.

6.3 Randomisation, blinding and treatment allocation

Before randomization, patients will be divided into risks-strata based on their individual risk on developing intraoperative hypotension. Computer-generated variable block randomization will be used to allocate patients (1:1) to either intervention or care-as-usual group, stratified for risk strata. Both the randomization and the assessment of the outcomes may be done by the same research team member. Hence, the assessment of the outcomes is officially not blinded. However, given the volume of participants and the elapsed time between randomization and outcome assessment, it is unlikely that the research team member remembers the randomization result for an individual patient participating in this study when assessing the outcomes at 30 days or six months after surgery. We will make sure that the randomization status is obscured from the assessors view within the workflow process. Patients are not actively blinded for the intervention, as they would be able to view their intraoperative medical record. Nonetheless, the result of the randomization will not actively be told to the patient. Caregivers cannot be blinded for the intervention due to the nature of the intervention strategy.

6.4 Study procedures

Screening of eligible patients will be done by screening the agenda of the preoperative assessment

clinic by a research team member using the in-/exclusion criteria specified in section 4.2 and 4.3. Eligible patients receive the study information at the preoperative assessment clinic on paper, using the patient information folder. In case it is not possible to give the patient the study information folder during his/her visit at the preoperative assessment clinic, for example because the consultation was done by telephone, then the patient will receive the study information folder by post. Before contacting patients outside the preoperative assessment clinic and providing them with the study information folder, the research member will always verify that the patient agreed to be contacted for medical research.

Patients who visit the preoperative assessment clinic will receive detailed explanation of the study procedures and questions will be answered. Before signing the informed consent form, the patient will be asked at all times whether they have be given enough time to consider participation in this study. If a patient wants to participate in this study, the informed consent form can be signed directly on the preoperative assessment clinic. Patients can only sign informed consent on the preoperative assessment clinic if a research team member is present that can answer all questions.

It is possible that the informed consent form cannot be signed during his/her visit at the preoperative assessment clinic, for example because the consultation was by telephone or the patient needed more time to consider participation. In this case the patient will be contacted by telephone by a research member to give additional study information and answer questions if necessary. If the patient decides to participate in the study they have to give verbal consent on the telephone and they have to complete the informed consent form which is attached to the patient information folder and send it to the researcher by post or bring it during hospital admission before surgery. The researcher will sign the informed consent form and send a copy of the signed informed consent form by post or the patient will receive a copy during admission. If the patient has already signed the informed consent form on the preoperative assessment clinic, they will receive a copy directly.

In case patients are not contacted for participation before admission to the hospital, they will be visited in the hospital by a research member to provide them with study information and ask informed consent. If patient want to participate they can sign the informed consent form and receive a copy on paper. Patients will always be asked if they had enough time to consider participation in this trial.

After receiving informed consent, participating patients will be randomized into the intervention or care-as-usual arm using the randomization tool in Castor EDC. The results of the randomization will be documented in the Electronic Health Record. The anaesthesiologist who is responsible for the patient during the surgery can see the result of the randomization and act accordingly using the risk-based intervention strategy as described in section 5.1.1 and 5.1.2. On the day of surgery the anesthesiologist will always verify that the patient is aware of participation in this trial. This way the anesthesiologist confirms that the informed consent form was not signed by another person without knowledge of the patient. If the patient is unaware of participation or does not want to participate anymore he or she will no longer be a participant in the trial and the patient will receive care-as-usual.

The anesthesiologist will make a notation in the electronic health record in case the patient is unaware of participation.

All inpatients, regardless of participating in this study, receive the WHODAS 2.0 and EuroQoL 5D-5L questionnaires three times (preoperative, 30 days and 6 months after surgery) as a part of the standard of care. Hence, the procedure of obtaining the questionnaires will not be described in this protocol. In the informed consent form we will ask permission to use the results of the questionnaires for this study.
The study team is aware that signing the informed consent form ideally takes place in person on the study site. However, patients should be provided with enough time to consider participating for this study so signing the informed consent on the preoperative assessment clinic will not always be possible. The time between the hospital admission and the start of the surgery is often very short, as most patients are admitted to the hospital on the day of their surgery, so it will not be achievable to obtain informed consent for all patients during admission. In addition, it is not desirable to request patients for an additional visit to the hospital to sign the informed consent form, especially during the current COVID-19 pandemic. Hence, we deem it the best option for our patients to create the possibility to sign the informed consent form at home and send it to the study site by mail, where it will be signed by a member of the research team. If the informed consent could not be obtained by the study site before surgery, the research team member will visit the patient on the day of hospital admission, to obtain informed consent. Only patients who have signed the informed consent form before surgery will enter the study. If patients decline study participation, they will receive perioperative care as usual.

A patient panel was consulted to discuss these study procedures. Especially the informed consent procedure as described above was preferred to offer patients enough time to consider participation and to minimize patient burden, i.e. to prevent patients from being asked to visit the hospital an extra time only to sign the informed consent form.

6.5 Withdrawal of individual subjects

Patients can leave the study at any time for any reason if they wish to do so without any consequences. The investigator can decide to withdraw a patient from the study for urgent medical reasons.

6.6 Replacement of individual subjects after withdrawal

Patients who withdraw before surgery (which may be before or after randomization) will be replaced by new study participants. If patients choose to withdraw from the follow up, this will most likely be

similar in both groups. Patients will not be replaced in these cases. The patients who withdraw will receive standard treatment. All data that has been collected until the moment the patient chose to withdraw will be used for analysis.

6.7 Follow-up of subjects withdrawn from treatment

No follow-up will occur if patients withdraw from the study. All data that has been collected until the moment the patient chose to withdraw will be used for analysis.

6.8 Premature termination of the study

As described in section 5.1.4 and 8.4, the decision to continue or terminate the trial will be made at the end of the adaptation phase (6 months after study initiation) and the decision whether to start the real world implementation will be made after the inclusion of 2500 patients (around study month 13). However, termination of the trial due to insufficient effectiveness of the intervention strategy will not be considered premature termination, rather a planned termination of the trial.
After the inclusion of 2500 patients, the effectiveness of the intervention strategy will be determined. In case the patients in the intervention arm show a 5%-point or more increase in functional disability after 30 days compared to the care-as-usual arm measured with the WHODAS 2.0 questionnaire, the study team will discuss whether or not to terminate the study prematurely.

6.9 The impact of COVID-19 on this study

Besides the possibility for premature termination based on the effectiveness of the intervention strategy, it’s also possible that the global COVID-19 crisis will affect the continuation of this trial. During the adaptation phase, an effective implementation of the intervention strategy is considered essential for the success of this trial. Input from both centres is necessary for this successful implementation Hence, if one of the participating centres needs to pause further enrollment due to the hospital policy or downscaling of elective procedures, both centres will stop enrolling new patients. When elective surgery can be resumed in both centres, the study can also enroll patients again. After the adaptation phase, a more pragmatic decision whether to continue or pause the trial can be made.

Thus, the enrollment of patients in this COVID-19 era is related to the number of elective surgeries performed and the policy during the COVID-19 in both hospitals. When no more elective surgeries will be performed, the team will gather a meeting and discuss possible solutions to continue with this trial. Amendments in the research protocol will be submitted to the METC if necessary.

6.10 Protocol deviation and violation

The aim of the intervention strategy is to maintain patients at a higher target blood pressure, not to instruct the anesthesiologist to make specific treatment decisions to reach the target blood pressure. The guidelines only provide suggestions and are not considered a treatment protocol with strict adherence. As the anesthesiologist remains autonomous in the chosen strategy for each individual patient, there are no formal violations and/or deviations from protocol.

7. SAFETY REPORTING

7.1 Temporary halt for reasons of subject safety

In accordance to section 10, subsection 4, of the WMO, the sponsor (UMC Utrecht) will suspend the study if there is sufficient ground that continuation of the study will jeopardise subject health or safety. The sponsor will notify the accredited METC without undue delay of a temporary halt including the reason for such an action. The study will be suspended pending a further positive decision by the accredited METC. The investigator will take care that all subjects are kept informed.

7.2 AEs, SAEs

7.2.1 Adverse events (AEs)

Adverse events are defined as any undesirable experience occurring to a subject during the study, whether or not considered related to the intervention strategy. All adverse events that subjects spontaneously report to the investigator or his staff, in addition to the adverse events that are observed by the investigator or his staff following data collection as described in section 6 will be recorded.

7.2.2 Serious adverse events (SAEs)

A serious adverse event is any untoward medical occurrence or effect that

- results in death;
- is life threatening (at the time of the event);
- requires hospitalisation or prolongation of existing inpatients' hospitalisation;
- results in persistent or significant disability or incapacity;
- is a congenital anomaly or birth defect; or
- any other important medical event that did not result in any of the outcomes listed above due to medical or surgical intervention but could have been based upon appropriate judgement by the investigator.

An elective hospital admission will not be considered as a serious adverse event.

All SAEs will be reported by the investigator to the sponsor after obtaining knowledge of the events. Only SAEs that are observed by the researcher following data collection as described in section 6 of this study protocol or SAEs that are spontaneously reported by the subject or a colleague to the researchers will be recorded.The sponsor will report the SAEs every year with a line listing to ToetsingOnline together with the annual progress report. Due to the fact that the intervention

strategy only applies to treatment that is continued throughout the anaesthetic case and the strategy only employs treatments options that are already routinely used in standard care, it is considered unnecessary to report SAEs more often than once a year.

7.3 Follow-up of adverse events

All procedure related or possibly procedure related AEs will be followed until they have abated, until a stable situation has been reached or till end of study. Depending on the event, follow-up may require additional tests or medical procedures as indicated, and/or referral to the general physician or a medical specialist. Procedure related or possibly procedure related SAEs need to be reported annually, as defined in the protocol.

7.4 Data Safety Monitoring Board (DSMB) / Safety Committee

This study will make use of an independent Data Safery Monitoring Board (DSMB). The responsibilities and operating procedures of the DSMB have been outlined in the DSMB charter (K5. DSMB plan PRETREAT).

For the complete DSMB plan, we refer to the attached document ‘K5. DSMB plan PRETREAT’.

8. STATISTICAL ANALYSIS

All patients randomly allocated to either the treatment strategy or care-as-usual group will be considered as comprising the intention-to-treat population for all primary and secondary analyses. Baseline characteristics of participants will be described using frequencies with percentages for categorical variables and means with standard deviations and/or medians with interquartile ranges for continuous variables, as appropriate. Multiple imputation will be performed in case of any missing data. We assume a patient dropout of 20% at 6 months follow up (see Table 2 Sample size and power calculation). Baseline characteristics of patients who did and did not respond at 6 months follow up will be compared to examine whether selective dropout occurred.

The aim of this study is to investigate whether a proactive blood pressure management strategy affects the incidence of IOH and thereby influences persistent disability six months after elective non-cardiac surgery. Different scenarios might occur regarding the final conclusion of this study (Table 2). In the first scenario, the proactive blood pressure management strategy will result in a lower incidence of IOH and consequently lead to less disability at six months compared to patients randomized to the reactive blood pressure management strategy. The proactive blood pressure management strategy is superior to the reactive strategy. The second scenario shows less hypotension, but no difference in disability meaning that disability is not affected by IOH. In that case, the trial is inconclusive. In scenario 3 and 4, the blood pressure management strategy will not result in less IOH and therefore, no inferences can be made on its effect on disability at six months.

Table 2. Description of different scenarios for the proactive blood pressure management strategy relative to the reactive blood pressure management strategy

Scenario	Incidence IOH	Disability	Conclusion
1	↓	↓	Superiority of proactive strategy
2	↓	↑ or =	Inconclusive
3	↑ or =	↓	Inconclusive
4	↑ or =	↑ or =	Inferiority of proactive strategy

This trial will be analyzed using a Bayesian framework.(25) Bayesian statistics provide a formal method for combining pre-existing information (i.e. prior probability) with data that are collected in the clinical trial into the analysis so that the current state of knowledge can be updated (i.e. posterior probability). Unless otherwise noted, all parameter estimates will be reported as means or medians with 95% credible intervals. All models will be fit using Markov Chain Monte Carlo (MCMC).

8.1 Primary study parameter

Mixed effects linear regression models will be fitted to analyze the effect of the proactive blood pressure management strategy on postoperative disability score as measured by WHODAS 2.0 at six months as a function of the randomization assignment, the assigned IOH risk stratum, and their interaction term. Random effects , including a random intercept, will be estimated to account for clustering effects within the participating centers (i.e. UMC Utrecht or AMC).

For this trial, prior probabilities are assigned non-informative for all included parameters. We set superiority as 0.95 or greater posterior probability that the treatment strategy lowers the disability score by ≥ 5%-point as measured by the WHODAS 2.0 compared to the care-as-usual group. Equivalence is set as 0.90 or greater posterior probability that the linear regression coefficient difference in disability score lies between 0 and 5%-point.

The possibility exists that a Hawthorne effect may occur.(26) As the anaesthesia team gets more experience with the intervention strategy over time, it is possible that they start applying (parts of) the intervention strategy to patients of the care-as-usual group, resulting in cross-contamination. The following situations will be seen as an indication for the presence of a Hawthorne effect; 1) a negligible difference in drug administration behaviour and blood pressures between the study groups; 2) a decreasing difference in drug administration behaviour and blood pressures between the study groups. When the Hawthorne effect turns out to be severe, the difference between study groups may be negligible even when the strategy works and improves patient outcome. If such an Hawthorne effect occurs, we will perform an additional interrupted time series analysis, comparing the behaviour, blood pressure management and disability scores of both groups within the randomized trial to a baseline period – i.e. measurements performed during the period preceding the start of the trial. Sensitivity analysis of the primary outcome will be performed using per protocol analysis.

8.2 Secondary study parameters

To assess whether the proactive blood pressure strategy indeed results in less IOH compared to usual care, the incidence of IOH (defined as a MAP <65 mmHg) will be investigated as a secondary outcome. Mixed effects logistic regression models similar to the primary analysis will be constructed to calculate the posterior probability of the incidence of IOH as a function of the randomization assignment, the assigned IOH risk stratum, and their interaction term. Again, prior probabilities are assigned non-informative for all included parameters at the first analysis. The first interim analysis will occur after three months after the inclusion of the first patient. After that first adaptive analysis, they

will be planned to be repeated every six weeks, perpetually, until 6 months after the first included patient. We set superiority as 0.95 or greater posterior probability that the treatment strategy lowers the incidence of IOH by ≥ 30% compared to the care-as-usual group. Equivalence is set as 0.90 or greater posterior probability of the reduction in the incidence of IOH lies between 0 and 30%. In case the risk-based intervention strategy is not able to reduce the relative risk of hypotension and/or show a clear increase in the intraoperative use of cardiovascular drugs, the study will end after the adaption phase (i.e. six months after study initiation).

8.3 Other study parameters

All other outcomes described in 6.2 will be analyzed using mixed effects regression models with appropriate link functions (e.g. linear, logistic or Poisson regression). Interim analyses are planned for all secondary outcomes on the process and behavior level (e.g. depth and duration of IOH, intraoperative use of cardiovascular drugs and blood loss) at the similar time points as the interim analyses for the incidence of IOH. No interim analyses are planned for the outcomes on the patient level.

8.4 Interim analysis

In the first six months of the trial, the risk-based interventions strategy will be evaluated and further revised in adaption cycles of three weeks, as described in section 5.1.4 (also see Appendix 3 for an overview of the study). Besides clinical insight obtained from the weekly interviews with members of the anaesthesia staff, descriptive data on the incidence, depth and duration of hypotension and hypertension, and cardiovascular drug use (dose, timing, type) will be available to quantify the impact of the risk-based interventions strategy.

First, it wil be checked whether the guidelines provided in the flowchart are put into practice by the anesthesia team members. In case the anesthesia team doesn’t follow the guidelines we will try to find out whether the anesthesia team does not agree with the guidelines that are provided or whether the advice is complex to implement into practice. Possible explanations for incompliance with the guidelines will be evaluated in weekly interviews with the members of the anesthesia staff. Adaptations will be made if necessary, for example by lowering the advised infusion speed at the start of the induction if the infusion speed is considered too high or simplify the guidelines in case the anesthesia team considers the guidelines to be too complex. If it turns out that the intervention strategy is not put into practice in specific patient groups the need to add contraindications will be evaluated. In order to increase the compliance and avoid complexity, it has been ensured that the guidelines are based on the current practice of anesthesia providers. The guidelines that will be

provided will keep the cumulative doses of perioperative drugs within the therapeutic limits considered to be safe.

Second, the effect of the guidelines on intraoperative blood pressures will be evaluated. The intervention is considered successful if it results in a 30% or more relative risk reduction (incidence of MAP<65 mmHg) of intraoperative hypotension. In case the intervention doesn't reduce the relative risk of intraoperative hypotension by 30% the intervention will be intensified for example by increasing the doses of vasopression. In the event that the intervention stategy leads to a higher incidence of possibly dangerous hypertension (MAP>130mmHg), we will reduce the infusion doses of the vasopressor.

The need for continuing the adaption cycles will be reflected by performing planned interim analyses at three and six months after study initiation as described in section 8.2. The intervention is considered successful if it results in a 30% or more relative risk reduction of intraoperative hypotension. Also the anesthesia providers should agree that the intervention strategy is easy to implement in practice and the reduction in hypotension should be accompanied by a clear change in intraoperative administration of cardiovascular drugs (e.g. timing, dose, type). In case the risk-based intervention strategy is not able to reduce the relative risk of hypotension by 30% the study will end after the adaption phase. When the intervention has a successful impact on the clinical process and no or little further progress is expected, the adaption cycles will end and only the evaluation of the effect of the treatment strategies will continue during the remaining part of the trial (see Appendix 3 for milestones of remaining part of the trial).

After inclusion of 2500 patients, we will perform an interim analysis to investigate the effect of the risk-based intervention strategy on the functional disability of patients at 30 days after surgery. The analyses are similar as previously described for the primary endpoint (section 8.1). If the overall reduction in functional disability at 30 days is 4%-point or greater, a 'real-world implementation' study will be started. During this real-world implementation study, we will evaluate the feasibility of the implementation of the intervention strategy in other hospitals, based on the behavioural data and blood pressure management data only. The real world implementation study is not a part of this trial and will not be described in this protocol. If the overall reduction in functional disability at 30 days is lower than 4%, the real world implementation phase will not start. If the intervention strategy leads to an increase of 5%-point or more on the WHODAS 2.0 at 30 days compared to the care-as-usual arm of the study, the study will terminate as described in section 6.8.

9. ETHICAL CONSIDERATIONS

9.1 Regulation statement

The study will be conducted according to the principles of the Declaration of Helsinki (July, 2018) and in accordance with the Medical Research involving Subjects Act (WMO). Patient will need to provide informed consent (IC) before randomization for the study will occur. All data will be handled according to the General Data Protection Regulation (GDPR, Algemene Verordening Gegevensbescherming in Dutch, (AVG)).

9.2 Recruitment and consent

The recruitment and consent procedures are described under section 6.4.

9.3 Benefits and risks assessment, group relatedness

Benefits:
With the proactive risk-based intervention strategy, a low blood pressure may be avoided. Avoiding intraoperative low blood pressure can possibly lead to less functional disability after surgery. Besides the positive effect of this intervention strategy for the patient, avoiding hypotension and possibly reducing complications after surgery might reduce the healthcare costs by millions of euros.

Burden:
The burden for participating patients is considered low. All interventions will take place whilst the patient is undergoing surgery. All patients undergoing intermediate/high risk procedures will receive the WHODAS 2.0 and EQ5D-5L questionnaires as part of usual care.

Risks:
It is important to explain that the physiology of patients undergoing elective surgery under general anesthesia is different from patients undergoing an emergency procedure, coming from the intensive care unit or residing at the nursing ward after surgery. Patients undergoing elective surgery are at high risk of developing hypotension as a result of loss of sympathetic tone, vasodilatation and myocardial depression due to the administration of anesthetics and analgesics. Under elective circumstances, all measures taken by the anesthesia team to maintain adequate blood pressure levels, for example by administrating fluids or vasopressors, serve to counteract the side effects induced by the anesthesia and surgery itself.

During urgent procedures or emergency situations, there are either major ‘mechanical’ alterations in a patient’s circulatory system (e.g. hemorrhage or heart failure), or an increased stress response that cause the low blood pressure or even circulatory shock. Specific events during an

elective procedure can cause similar mechanical alterations, resulting in an emergency situation that are beyond the scope of the intervention of this study.

Tissue injury causes a stress response. This stress response activates the sympathetic nervous system and the endocrine system, increasing circulatory (heart rate, blood pressure), respiratory parameters (respiratory rate) and cortisol levels. In addition, the stress response induces a systemic inflammatory reaction. Although the stress response is a very useful defensive mechanism of the body for restoring tissue injury and fighting infection, it is also a major cause of perioperative adverse effects such as acute kidney injury, myocardial injury, and perioperative infections.(27)However, the main aim of anesthesia is to attenuate that stress response, especially during the surgical procedure. That is why under elective conditions, an elevated stress response that results in atrial fibrillation or circulatory shock do not occur unless a specific event has triggered it (e.g. anaphylaxis). When such a specific event occurs this thus becomes an emergency situation that is beyond the scope of the intervention of this study, and for which other specific clinical guidelines exist are present as emergency checklists in the operating room.

The clinical guideline suggests how to use vasopressor therapy and when to consider other options such as fluid therapy or chronotropes. The clinical guideline only includes cardiovascular drugs that are part of regular anesthesia practice, and the suggested dosages are well in the lower dosage range for the vasopressor drugs. For example, the anesthesiologist starts with a norepinephrine infusion for a 60 kg patient. Because of hypotension she increases the dosage three times without the intended effect. The protocol suggests not to further increase the vasopressor dosage (see Appendix 2). If other interventions do not help, so the anesthesiologist may decide to accept a lower blood pressure. For this patient the norepinephrine dosage range has been 0.05-0.15 mcg/kg/min. In contrast, in ICU patients 0.15 mcg/kg/min is at the lower end of the dosage spectrum, with dosages of 0.5-1.0 mcg/kg/min to be considered high dosages.

A comparable single-center pilot study of patients undergoing elective noncardiac surgery called the HYPE trail, performed in the Amsterdam UMC, showed that the use of a machine learning–derived early warning system with a similar proactive treatment protocol resulted in less intraoperative hypotension. Importantly, the decrease of intraoperative hypotension was not accompanied by the administration of higher cumulative doses of medication or fluids and the amount of possibly dangerous intraoperative hypertension (MAP > 130 mmHg) was not increased. This can be explained by the fact that a proactive treatment of hypotension will result in the administration of more frequent small doses of medication resulting in less fluctuations of the blood pressure. Previous literature suggests that less blood pressure fluctuations are associated with better postoperative outcomes.(28) Hence, the available literature suggests that a proactive treatment strategy for the treatment of intraoperative hypotension is safe and possibly even superior compared to a reactive treatment of intraoperative hypotension. Some specific procedures, for example obstetric and neurosurgery

procedures, already use a proactive treatment strategy to prevent hypotension. Higher prevalence of adverse events due to the proactive treatment strategy in these specific patient groups is not described..

To improve the safety of the intervention strategy even further the decision was made to provide guidelines in the form of a medical protocol instead of a fixed protocol that should be followed at all times. The anaesthesia team continuously monitors the patient during the procedure - including the blood pressure – and are at complete liberty to intervene if they believe a different blood pressure strategy is in the best interest of the patient. In addition, we will closely monitor how the strategy works out on intraoperative blood pressure management during the adaptive phase of the trial and adjust the proactive strategy or the clinical guideline if necessary (see section 8.4). However, complications due to the intervention strategy cannot be completely excluded. Hence, the risk of participating in this study is moderate.

9.4 Compensation for injury

Each participating centre/investigator has a liability insurance which is in accordance with article 7 of the WMO.

The sponsor (also) has an insurance which is in accordance with the legal requirements in the Netherlands (Article 7 WMO). This insurance provides cover for damage to research subjects through injury or death caused by the study. The insurance applies to the damage that becomes apparent during the study or within four years after the end of the study.

9.5 Incentives

Patients won’t receive incentives when participating in this study.

10. ADMINISTRATIVE ASPECTS, MONITORING AND PUBLICATION

10.1 Handling and storage of data and documents

The data will be handled confidentially. The patient data will be retrieved from the electronic medical record systems through the local enterprise data warehouses and the Castor Electronic Data Capture server. All data will be pseudonymized before extraction for further analysis. These data will be stored on an encrypted storage drive within the local hospital network, at an appropriate sub-directory and will only be accessible by key study personnel named in the site signature and delegation log. Besides the key study personnel, study monitors/auditors have access to identifiable data. Data will be kept for 15 years. Any data that leaves the hospitals premises will be de-identified. The randomization key is kept at the local hospital. Any publication arising from this study will not contain data that can be traced back to individual patients.

The Principal investigator is responsible for handling and storage of data and documents on their side. For more information we refer to the latest data management plan, attached as a separate document.

10.2 Monitoring and Quality Assurance

The monitoring will be performed by the an external monitor from Julius Clinical Research BV. Details on the monitoring process are described in the monitoring plan. A risk assessment was performed using the ‘Hulplijst Risicoclassificatie’ by the NFU.(29) The final risk classification was ‘Moderate’.

10.3 Amendments

Amendments are changes made to the research after a favorable opinion by the accredited METC has been given. All amendments will be notified to the METC that gave a favorable opinion.

10.4 Annual progress report

The sponsor/investigator will submit a summary of the progress of the trial to the accredited METC once a year. Information will be provided on the date of inclusion of the first subject, numbers of subjects included and numbers of subjects that have completed the trial, serious procedure related adverse events/adverse event line listings, other problems, and amendments.

10.5 Temporary halt and (prematurely) end of study report

The investigator/sponsor will notify the accredited METC of the end of the study within a period of 8 weeks. The end of the study is defined as the last patient's six months postoperative questionnaire in on the last active participating centre (patient 5000).

The sponsor will notify the METC immediately of a temporary halt of the study, including the reason of such an action.

In case the study is ended prematurely, the sponsor will notify the accredited METC within 15 days, including the reasons for the premature termination.

Within one year after the end of the study, the investigator/sponsor will submit a final study report with the results of the study, including any publications/abstracts of the study, to the accredited METC.

10.6 Public disclosure and publication policy

All study sides remain the owner of their own study data. As the clinical study is a multi-centre study, any publication based on the results obtained at the study site shall not be made before the first multi-centre publication or presentation, which shall be coordinated by the Sponsor. If a multi-centre publication is not published within twelve months after completion of the clinical study and lock of the clinical study database at all research sites that are part of the multi-centre studie or any earlier termination or abandonment of the study, the site investigator and/or members of the research staff shall have the right to publish or present the methods and results of the clinical study. All study sites are entitled to examine the manuscript prior to publication and to make comments on it.

11. REFERENCES

1. Centraal Bureau voor de Statistiek. CBS Statline - Operaties in het ziekenhuis. 2014.
2. Wesselink EM, Kappen TH, Torn HM, Slooter AJC, van Klei WA. Intraoperative hypotension and the risk of postoperative adverse outcomes: a systematic review. Br J Anaesth. 2018 Oct;121(4):706–21.
3. Bijker JB, van Klei WA, Kappen TH, van Wolfswinkel L, Moons KGM, Kalkman CJ. Incidence of intraoperative hypotension as a function of the chosen definition: literature definitions applied to a retrospective cohort using automated data collection. Anesthesiology. 2007 Aug;107(2):213–20.
4. Salmasi V, Maheshwari K, Dongsheng Y, Mascha EJ, Singh A, Sessler DI, et al. Thresholds , and Acute Kidney and Myocardial Injury after Noncardiac Surgery A Retrospective Cohort Analysis. Anesthesiology. 2017;126(1):47–65.
5. Sessler DI, Bloomstone JA, Aronson S, Berry C, Gan TJ, Kellum JA, et al. Perioperative Quality Initiative consensus statement on intraoperative blood pressure, risk and outcomes for elective surgery. Br J Anaesth. 2019;
6. Ustün TB, Chatterji S, Kostanjsek N, Rehm J, Kennedy C, Epping-Jordan J, et al. Developing the World Health Organization Disability Assessment Schedule 2.0. Bull World Health Organ. 2010 Nov;88(11):815–23.
7. Shulman MA, Myles PS, Chan MT V, McIlroy DR, Wallace S, Ponsford J. Measurement of disability-free survival after surgery. Anesthesiology. 2015 Mar;122(3):524–36.
8. Myles P, Bellomo R, Corcoran T, Forbes A, Wallace S, Peyton P, et al. Restrictive versus liberal fluid therapy in major abdominal surgery (RELIEF): rationale and design for a multicentre randomised trial. BMJ Open. 2017;7(3):e015358.
9. Herdman M, Gudex C, Lloyd A, Janssen M, Kind P, Parkin D, et al. Development and preliminary testing of the new five-level version of EQ-5D (EQ-5D-5L). Qual Life Res. 2011;
10. van Waes JAR, van Klei W a, Wijeysundera DN, van Wolfswinkel L, Lindsay TF, Beattie WS. Association between Intraoperative Hypotension and Myocardial Injury after Vascular Surgery. Anesthesiology. 2016 Jan;124(1):35–44.
11. NVA. NVA Doelmatigheidsagenda klinisch evaluatie-onderzoek. Utrecht; 2018.
12. Futier E, Lefrant J-Y, Guinot P-G, Godet T, Lorne E, Cuvillon P, et al. Effect of Individualized vs Standard Blood Pressure Management Strategies on Postoperative Organ Dysfunction Among High-Risk Patients Undergoing Major Surgery: A Randomized Clinical Trial. JAMA. 2017;318(14):1346–57.

13. Sun Y, Chai F, Pan C, Romeiser JL, Gan TJ. Effect of perioperative goal-directed hemodynamic therapy on postoperative recovery following major abdominal surgery-a systematic review and meta-analysis of randomized controlled trials. Crit Care. 2017;21(1):1–17.
14. Panjasawatwong K, Sessler DI, Stapelfeldt WH, Mayers DB, Mascha EJ, Yang D, et al. A Randomized Trial of a Supplemental Alarm for Critically Low Systolic Blood Pressure. Anesth Analg. 2015 Dec;121(6):1500–7.
15. McCormick PJ, Levin MA, Lin H, Sessler DI, Reich DL. Effectiveness of an Electronic Alert for Hypotension and Low Bispectral Index on 90-day Postoperative Mortality. Anesthesiology. 2017;126(6):1113–20.
16. Kappen TH, Van Loon K, Kappen MAM, Van Wolfswinkel L, Vergouwe Y, Van Klei WA, et al. Barriers and facilitators perceived by physicians when using prediction models in practice. J Clin Epidemiol. 2016;70.
17. Kappen TH, van Klei WA, van Wolfswinkel L, Kalkman CJ, Vergouwe Y, Moons KGM. Evaluating the impact of prediction models: lessons learned, challenges and recommendations. Diagnostic Progn Res. 2018;[in press].
18. Futier E, Lefrant JY, Guinot PG, Godet T, Lorne E, Cuvillon P, et al. Effect of individualized vs standard blood pressure management strategies on postoperative organ dysfunction 6among high-risk patients undergoing major surgery: A randomized clinical trial. JAMA - J Am Med Assoc. 2017;
19. Toronto, Ontario CUHN. Clinical Characteristics and Functional Outcomes of Postoperative Myocardial Injury: a Prospective Cohort Study. TEAMS (Troponin Elevation After Major Surgery) Study [Internet]. 2014. Available from: Clinicaltrials.Gov
20. R Core Development Team. R: A language and environment for statistical computing. Vienna, Austria. 2019;
21. Shulman MA, Myles PS, Chan MTV, McIlroy DR, Wallace S, Ponsford J. Measurement of disability-free survival after surgery. Anesthesiology. 2015;
22. Moonesinghe, S.R., et al., Systematic review and consensus definitions for the Standardised Endpoints in Perioperative Medicine initiative: patient-centred outcomes. Br J Anaesth. 2019; 123(5):664-670.
23. Clavien PA, Barkun J, de Oliveira ML, Vauthey JN, Dindo D, Schulick RD et al. The Clavien-Dindo classification of surgical complications: five-year experience. Ann Surg. 2009 Aug;250(2):187-96.

24. DICA. Databases jaaroverzicht 2016 [Internet]. 2016 Available from: https://dica.nl/jaarrapportage-2016/over-dica#dica-in-2016nbsp.
25. Koch KR. Introduction to bayesian statistics. Introduction to Bayesian Statistics. 2007.
26. De Amici D, Klersy C, Ramajoli F, Brustia L, Politi P. Impact of the Hawthorne effect in a longitudinal clinical study: The case of anesthesia. Control Clin Trials. 2000;
27. Manou-Stathopoulou V, Korbonits M, Ackland GL. Redefining the perioperative stress response: a narrative review. British Journal of Anaesthesia. 2019.
28. Hirsch J, DePalma G, Tsai TT, Sands LP, Leung JM. Impact of intraoperative hypotension and blood pressure fluctuations on early postoperative delirium after non-cardiac surgery. Br J Anaesth. 2015;
29. NFU. Richtlijn Kwaliteitsborging Mensgebonden Onderzoek 2019 [Internet]. 2019. Available from: https://www.nfu.nl/img/pdf/19.1360_Richtlijn_kwaliteitsborging_mensgebonden_onderzoek_2019.pdf

12. APPENDICES

12.1 Appendix 1: risk score to determine target blood pressure

The case-mix of the patients in the participating centres can vary over time, so the need to update the risk score will be reflected every six months during the trial. Hence the risk score shown below serves only as an example. Both centres will use their own risk score adjusted to the case-mix of the participating centre.

Stap 1: calculate thee amount of points

Points = (age / 5) + expected surgery time in hours

Stap 2: Correct for the type of anesthesia

General anesthesia:	points + 0
General anesthesia + epidural:	points + 3
Spinal anesthesia:	points - 6

Stap 3: Determine the category based on the surgical specialism

Category 1: neurosurgery, oncological ENT surgery, vascular surgery

Category 2: ENT surgery, orthopedic surgery, oncological gastrointestinal surgery, general surgery, traumasurgery, oncological gynaecology, oral and maxillofacial surgery

Category 3: fertility surgery, general gynaecology, plastic surgery, urology

Stap 4: Determine the risk score based on the ASA-class, category and points

Low risk	Intermediate	High risk
Category 2 + ASA 1 + points < 13	All other patients	Category 1 + ASA 1 + points > 18
Category 2 + ASA 2 + points < 10		Category 1 + ASA 2 + points > 16
Category 3 + ASA 1 + points < 14		Category 1 + ASA 3/4 + points > 13
Category 3 + ASA 2 + points < 11		Category 2 + ASA 1 + points > 21
		Category 2 + ASA 2 + points > 19
		Category 2 + ASA 3/4 + points > 16
		Category 3 + ASA 1 + points > 22
		Category 3 + ASA 2 + points > 20
		Category 3 + ASA 3/4 + points > 17

12.2 Appendix 2 Guidelines to achieve target blood pressure

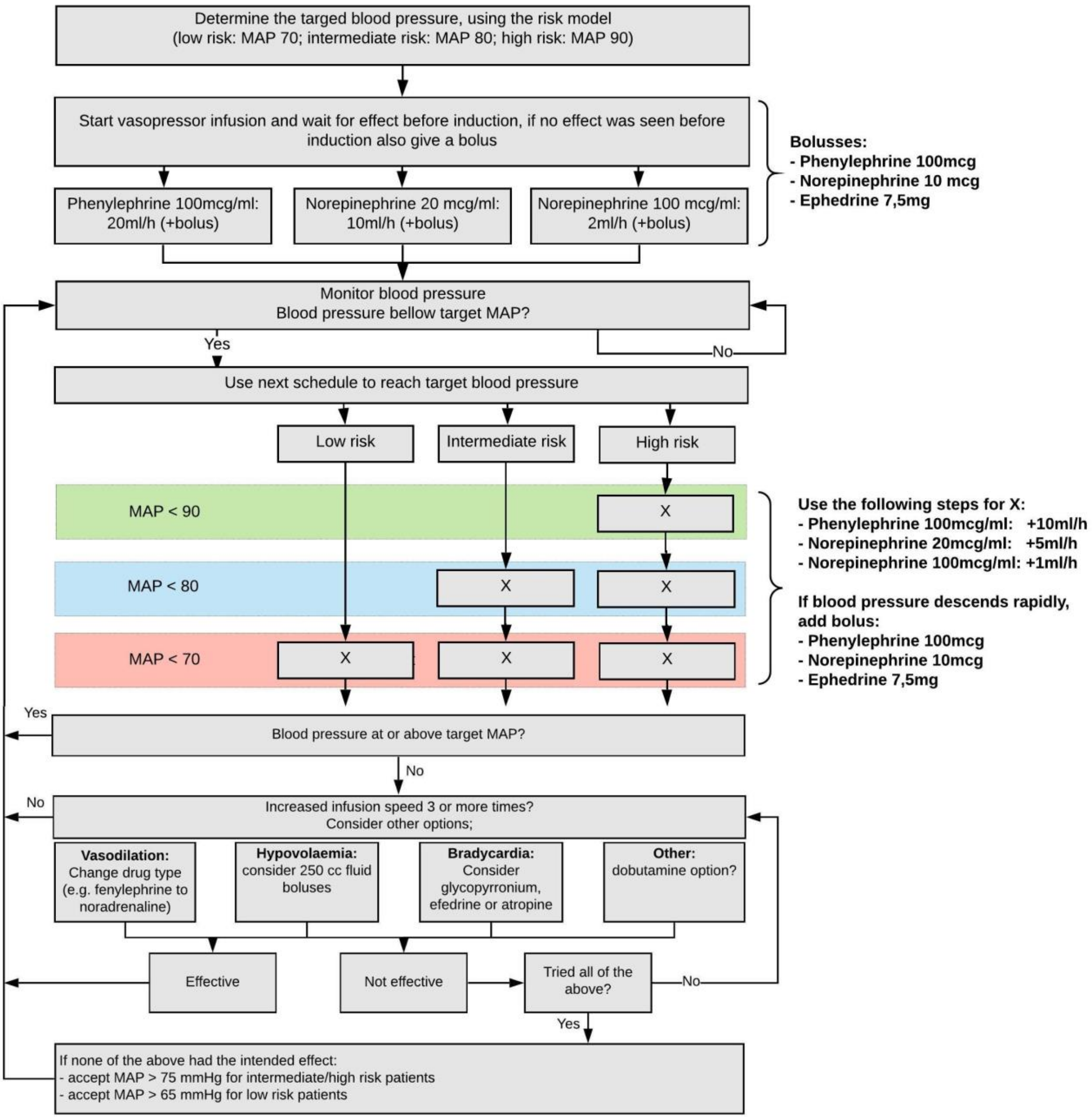

12.3 Appendix 3 Overview of the different study phases

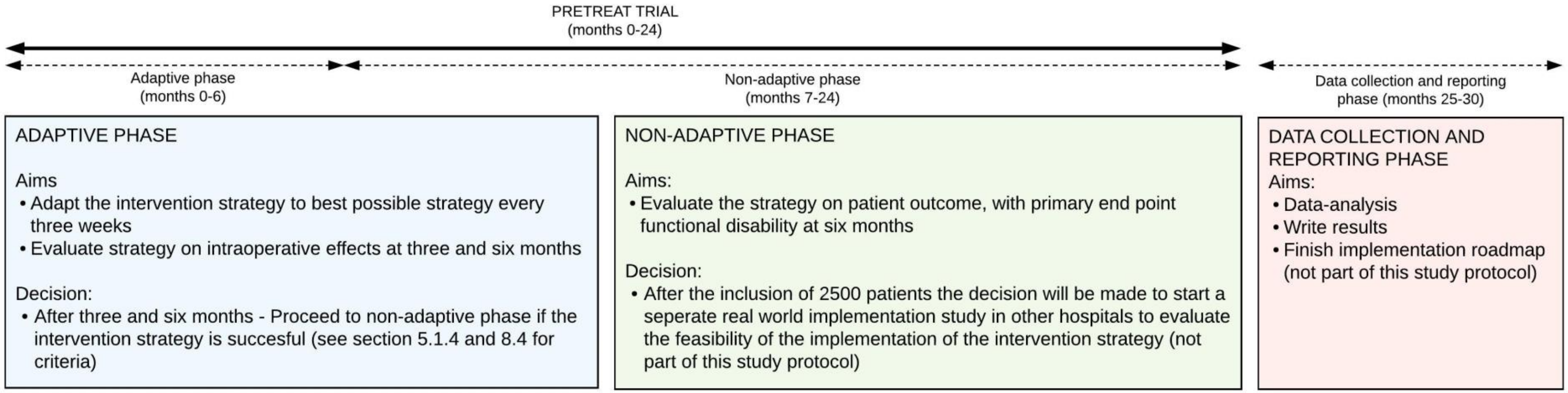

Supplement: Multimedia component 1 [file mmc1.pdf]
