# Supplementary material for: The effect of proactive versus reactive treatment of hypotension on postoperative disability and outcome in surgical patients under anaesthesia (PRETREAT): clinical trial protocol and considerations
Source: BJA Open. 2024 Feb 29;9:100262. doi: 10.1016/j.bjao.2024.100262 (PMC10910055; doi:10.1016/j.bjao.2024.100262)
Supplement: Multimedia component 3 [file mmc3.docx]

**Figure 1. Consort flowchart**

Excluded (n= 40.000)

♦  Not meeting inclusion criteria – (n= 37.000)

♦  Declined to participate (n= 1.500)

♦  Other, e.g. logistics (n= 1.500)

Assessed for eligibility (n=45.000)

## Enrollment

Analysed (n= 2.100 )

Analysed (n= 2.100 )

Lost to follow-up – consent withdrawn or unable to contact to complete survey (n= 400)

Lost to follow-up – consent withdrawn or unable to contact to complete survey (n= 400)

Allocated to usual care (n= 2.500)

♦ Received allocated intervention (n= 2.500)

♦ Did not receive allocated intervention – medical decision (n= 0)

Allocated to intervention (n= 2.500)

♦ Received allocated intervention (n= 2.500)

♦ Did not receive allocated intervention – medical decision (n= 0)

## Allocation

## Follow-Up

## Analysis

Randomized (n= 5.000 )
